# A harmonized public resource of deeply sequenced diverse human genomes

**DOI:** 10.1101/2023.01.23.525248

**Published:** 2023-02-02

**Authors:** Zan Koenig, Mary T. Yohannes, Lethukuthula L. Nkambule, Julia K. Goodrich, Heesu Ally Kim, Xuefang Zhao, Michael W. Wilson, Grace Tiao, Stephanie P. Hao, Nareh Sahakian, Katherine R. Chao, gnomAD Project Consortium, Michael E. Talkowski, Mark J. Daly, Harrison Brand, Konrad J. Karczewski, Elizabeth G. Atkinson, Alicia R. Martin

**Affiliations:** 1 Stanley Center for Psychiatric Research, The Broad Institute of MIT and Harvard, Cambridge, MA 02142, USA; 2 Analytic and Translational Genetics Unit, Massachusetts General Hospital, Boston, MA 02114, USA; 3 Program in Medical and Population Genetics, The Broad Institute of MIT and Harvard, Cambridge, MA 02142, USA; 4 Center for Genomic Medicine, Massachusetts General Hospital, Boston, MA 02114, USA; 5 Department of Neurology, Massachusetts General Hospital and Harvard Medical School, Boston, MA 02114, USA; 6 Broad Genomics, The Broad Institute of MIT and Harvard, 320 Charles Street, Cambridge, MA, 02141, USA; 7 Institute for Molecular Medicine Finland, Helsinki, Finland; 8 Department of Molecular and Human Genetics, Baylor College of Medicine, Houston, TX, 77030, USA

## Abstract

Underrepresented populations are often excluded from genomic studies due in part to a lack of resources supporting their analysis. The 1000 Genomes Project (1kGP) and Human Genome Diversity Project (HGDP), which have recently been sequenced to high coverage, are valuable genomic resources because of the global diversity they capture and their open data sharing policies. Here, we harmonized a high quality set of 4,096 whole genomes from HGDP and 1kGP with data from gnomAD and identified over 155 million high-quality SNVs, indels, and SVs. We performed a detailed ancestry analysis of this cohort, characterizing population structure and patterns of admixture across populations, analyzing site frequency spectra, and measuring variant counts at global and subcontinental levels. We also demonstrate substantial added value from this dataset compared to the prior versions of the component resources, typically combined via liftover and variant intersection; for example, we catalog millions of new genetic variants, mostly rare, compared to previous releases. In addition to unrestricted individual-level public release, we provide detailed tutorials for conducting many of the most common quality control steps and analyses with these data in a scalable cloud-computing environment and publicly release this new phased joint callset for use as a haplotype resource in phasing and imputation pipelines. This jointly called reference panel will serve as a key resource to support research of diverse ancestry populations.

## Introduction

The 1000 Genomes Project (1kGP) and Human Genome Diversity Project (HGDP) have been among the most valuable genomic resources because of the breadth of global diversity they capture and their open sharing policies with consent to release unrestricted individual-level data ^1–5^. Consequently, genetic data from these resources have been routinely generated using the latest genomics technologies and serve as a ubiquitous resource of globally diverse populations for a wide range of disease, evolutionary, and technical studies. These projects are complementary; the 1000 Genomes Project is larger and has consisted of whole genome sequencing (WGS) data for many years; as such, it has been the default population genetic reference dataset, consisting of 3,202 genomes including related individuals that were recently sequenced to high coverage ^6,7^. The 1000 Genomes Project has also been the most widely used haplotype resource, serving as a reference panel for phasing and imputation of genotype data for many genome-wide association studies (GWAS)^8,9^. HGDP was founded three decades ago by population geneticists to study human genetic variation and evolution and was designed to span a greater breadth of diversity, though with fewer individuals from each component population ^10,11^. Originally assayed using only GWAS array data, the 948 individuals have recently undergone deep WGS and fill some major geographic gaps not represented in the 1000 Genomes Project, for example in the Middle East, sub-Saharan Africa, parts of the Americas, and Oceania ^1^.

The 1kGP and HGDP datasets have been invaluable separately, but far larger genomic data aggregation efforts, such as gnomAD ^12^ and TOPMed ^13^, have clearly demonstrated the utility of harmonizing such datasets through the broad uptake of their publicly released summaries of large numbers of high-quality whole genomes. For example, the gnomAD browser of allele frequencies has vastly improved clinical interpretation of rare disease patients worldwide ^14^. Additionally, the TOPMed Imputation Server facilitates statistical genetic analyses of complex traits by improving phasing and imputation accuracy compared to existing resources ^13^. Yet, without individual-level data access from these larger resources due to more restrictive permissions, the 1kGP and HGDP genomes remain the most uniquely valuable resources for many of the most common genetic analyses. These include genetic simulations, ancestry analysis including local ancestry inference ^15^, genotype refinement of low-coverage genomes ^16^, granular allele frequency comparisons at the subcontinental level, investigations of individual-level sequencing quality metrics, and many more.

Previously, researchers wishing to combine HGDP and 1kGP into a merged dataset were left with suboptimal solutions. Specifically, the sequenced datasets had been called separately, requiring intersection of previously called sites rather than a harmonized joint-callset. Additionally, they were on different reference builds, requiring lifting over of a large dataset prior to merging, which introduces errors and inconsistencies. Here, we have created a best-in-class publicly released harmonized and jointly called resource of HGDP+1kGP on GRCh38 that will facilitate analyses of diverse cohorts. This globally-representative haplotype resource better captures the breadth of genetic variation across diverse geographical regions than previous component studies. Specifically, we aggregated these genomes into gnomAD and then jointly processed these 4,096 high-coverage whole genomes; jointly called variants consisting of single nucleotide variants (SNVs), insertions/deletions (indels), and structural variants (SVs); conducted harmonized sample and variant QC; and separately released these individual-level genomes to facilitate a wide breadth of analyses. We quantify the number of variants identified in this new callset compared to existing releases and identify more variants as a result of joint variant calling; construct a resource of haplotypes for use as a phasing and imputation panel; examine the ancestry composition of this diverse set of populations; and publicly release these data without restriction alongside detailed tutorials illustrating how to conduct many of the most common genomic analyses.

## Results

### A harmonized resource of high-quality, high coverage diverse whole genomes

**Figure 1 |:**
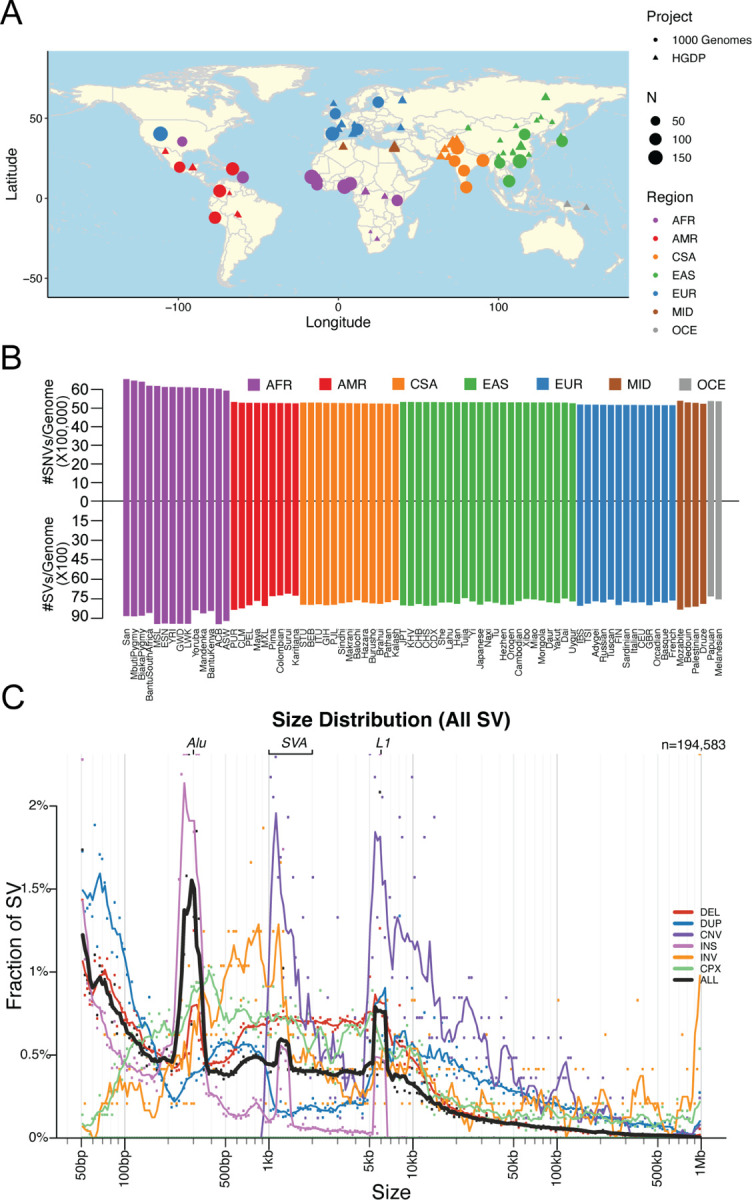
Geographical locations and genetic variants across populations. A) Global map indicating approximate geographical locations where samples were collected. Coordinates were included for each population originating from the Geography of Genetic Variants browser as well as meta-data from the HGDP ^1,21^. B) Mean number of SNVs versus SVs per individual within each population. Colors are consistent with geographical/genetic regions in A-B), as follows: AFR=African, AMR=admixed American, CSA=Central/South Asian, EAS=East Asian, EUR=European, MID=Middle Eastern, OCE=Oceanian. C) Sizes of SVs decay in frequency with increasing size overall with notable exceptions of mobile elements, including Alu, SVA, and LINE1. Abbreviations are deletion (DEL), duplication (DUP), copy number variant (CNV), insertion (INS), inversion (INV), or complex rearrangement (CPX).

Here, we have developed a high-quality resource of diverse human genomes for full individual-level public release along with a guide for conducting the most common genetic analyses. To this end, we first harmonized project meta-data and jointly called variants from 4,150 whole genomes recently sequenced to high coverage from the 1kGP and HGDP into gnomAD (Table S1) ^1,7^, the latter of which are new to gnomAD. Figure 1A shows the locations and sample sizes of populations included in this harmonized resource. After sample and variant QC ^17^ including ancestry outlier removal (Table S2, Methods), we identified 159,795,273 high-quality variants across 4,096 individuals, 3,378 of whom are inferred to be unrelated (Methods, Table S3). We computed the mean coverage within each population and project (Figure S1–2) as well as the mean number of SNVs per individual within each population to better understand data quality and population genetic variation (Table S4). While coverage was more variable among samples in HGDP (μ=34, σ=6, range=23–75X) than in 1kGP (μ=32, σ=3, range=26–66X), consistent with older samples and more variable data generation strategies ^1^, all genomes had sufficient coverage to perform population genetic analysis. Consistent with human population history and as seen before ^4^, African populations had the most genetic variation with 6.1M SNVs per individual, while out-of-Africa populations had an average of 5.3M SNVs SNVs (Table S4, Figure 1B). The San had the most genetic variants as well as singletons per genome on average overall (Table S4).

We generated a jointly genotyped structural variants (SVs) callset with the HGDP genomes and high-coverage 1kGP genomes ^7^ using the ensemble SV detection tool, GATK-SV ^18^ (Figure S3). In total, we identified 196,173 SV loci across all 4,150 HGDP and 1kGP samples. We detected a median of 8,123 SVs in each genome consisting primarily of deletions, duplications, and insertions (Figure 1). As expected, the frequencies of SVs were consistent with Hardy-Weinberg Equilibrium (Figure S4), and distributions matched expectations from previous cohorts with the vast majority of SVs being rare (84.2% SVs are <1% allele frequency among population). Additionally, SV size is inversely correlated with frequency ^7,18,19^, with notable exceptions of peaks consistent with known mobile elements, including ALU, LINE1, and SVA (Figure 1). Consistent with shorter genetic variation, we observed a higher frequency of SVs in African populations. The quality of our variant call sets have been evaluated using both the short-read and long-read WGS data generated by the 1kGP and the human genome structural variation consortium (HGSVC, ^7,20^). High precision was observed in the SV call set–among the 34 overlapping samples, 91.9% of the SVs were overlapped by either a short-read or long-read variant in the matched genome; the highest precision (97.6%) was observed for deletions followed by insertions (91.4%) and duplications (89.3%) (Table S6). We observed some differences in number SVs across samples from HGDP and 1kGP due to technical data generation differences, such as PCR status (Figure S6).

**Figure 2 |:**
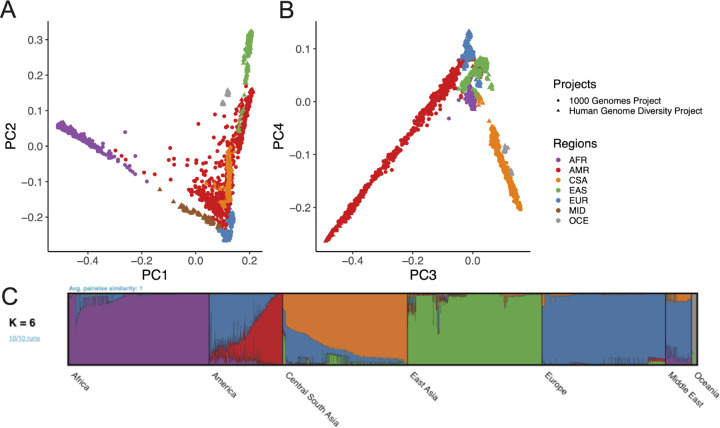
Global ancestry analysis of genetic structure in the HGDP and 1kGP resource. Regional abbreviations are as follows: AFR=African, AMR=admixed American, CSA=Central/South Asian, EAS=East Asian, EUR=European, MID=Middle Eastern, OCE=Oceanian. A-B) Principal components analysis (PCA) plots for A) PC1 versus PC2 and B) PC3 versus PC4 showing global ancestry structure across HGDP+1kGP. Subsequent PCs separated structure within geographical/genetic regions (Figure S9). C) ADMIXTURE analysis at the best fit value of K=6.

We examined global population genetic variation using principal component analysis (PCA) of the harmonized HGDP and 1kGP resource (Figure 2). As expected, we find PC1 differentiates AFR and non-AFR populations, PC2 differentiates EUR and EAS populations, and PC3–4 differentiate AMR and CSA populations. Subcontinental structure is also apparent in later PCs and within genetic regions, which we define as group meta-data labels in HGDP+1kGP (Table S1, Figure S7) roughly according to continental region. These results are recapitulated with the likelihood model implemented in ADMIXTURE, where K=2 identifies similar structure in PC1, K=3 identifies similar structure in PC2, and so on (Figure S7). The best fit value of K=6 shown in Figure 2 was chosen based on 5-fold cross-validation error (Figure S8).

### Population genetic variation within and between subcontinental populations

We investigated the ancestry composition of populations within harmonized meta-data labels (AFR, AMR, CSA, EAS, EUR, MID, and OCE; Table S1) using principal component analysis (PCA) and ADMIXTURE analysis. Subcontinental PCA highlights finer scale structure within geographical/genetic regions (Figure S9). For example, within the AFR, the first several PCs differentiate populations from South and Central African hunter-gatherer groups from others, then differentiate populations from East and West Africa. For AFR and AMR populations, individuals cluster similarly to the global PCA, reflecting some global admixture present in these populations. The MID and OCE populations are only made up of samples from the HGDP dataset, as 1kGP did not contain samples from these regions.

**Figure 3 |:**
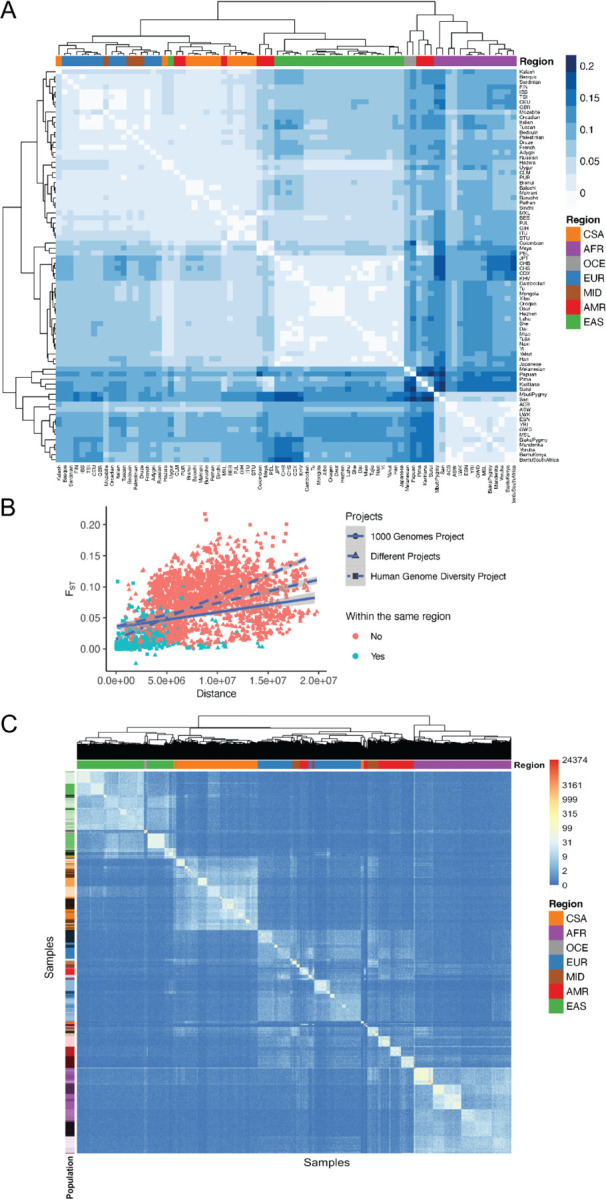
Genetic differentiation measured using common variants with F_ST_ and rare variants via *f*_*2*_ analysis and relationship with geography. A) F_ST_ heatmap illustrating genetic divergence between pairs of populations. B) Genetic differentiation measured by F_ST_ versus geographical distance in meters. C) Heatmap of *f*_*2*_ comparisons of doubleton counts between pairs of individuals. Column colors at the leaves of the dendrogram show colors corresponding to meta-data genetic region, while row colors correspond to population. Color bar indicates the number of doubletons shared across pairs of individuals, with more doubletons shared among individuals within the same population and genetic region versus across populations and genetic regions.

We measured population genetic differentiation using common variants with Wright’s fixation index, F_ST_ (Figure 3). When populations are clustered according to pairwise F_ST_ between groups, they largely cluster by geographical/genetic region labels with a few exceptions. For example, AMR populations are interspersed with other populations, consistent with having variable ancestry proportions that span multiple continents. Additionally, the MID populations are interspersed among the EUR populations. We also compared F_ST_ versus geographical distance, recapitulating previous work showing a linear relationship ^22^, but also showing that there are differences by project; specifically, HGDP has a steeper slope relating distance to F_ST_ (Figure 3), likely reflecting the anthropological design intended to capture more divergent populations compared to the samples in 1kGP that reflect some of the largest populations.

F_ST_ measurements require group comparisons and are only based on common variants, which typically arose early in human history. We also compared rare variant sharing via pairwise doubleton counts (*f*_*2*_ analyses, Figure 3). On average, pairs of individuals within a population share 51.83 doubletons, although this varies considerably as a function of demography. For example, due to the elevated number of variants in individuals of African descent (Figure 1), pairs of individuals within AFR populations share on average 76.38 doubletons, whereas pairs of individuals within out-of-Africa populations share 43.74 doubletons. Very few doubletons are shared among pairs of individuals across populations within a geographical/genetic region (mu=6.79, sd=18.31), and even fewer are shared among pairs of individuals across populations from different genetic regions (mu=0.8, sd=1.78). *f*_*2*_ clustering tends to follow project meta-data labels by geographical/genetic region, with a few exceptions.

### A catalog of known versus novel genomic variation compared to existing datasets

**Figure 4 |:**
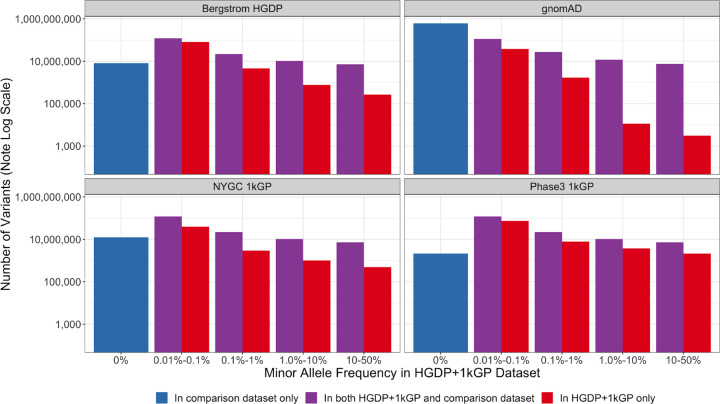
Number of variants identified in this dataset compared to commonly used existing datasets as a function of allele frequency. The number of variants on a log scale is plotted by minor allele frequency bin within the harmonized HGDP+1kGP dataset. We show variants found in the harmonized HGDP+1kGP dataset only (red), variants shared between the harmonized dataset and each comparison dataset (purple), and variants that are only found in each comparison dataset (blue).

To demonstrate the added benefit of jointly calling these two datasets, we have compiled metrics that compare our harmonized dataset with each individual dataset comprising it ^1,7^, the previous phase 3 1kGP dataset sequenced to lower coverage ^4^, and the widely used gnomAD dataset ^17^. This jointly called HGDP+1kGP dataset contains 159,795,273 SNVs and indels that pass QC, whereas phase 3 1kGP has 73,257,633, high-coverage WGS of 1kGP (referred to here as NYGC 1kGP based on where they were sequenced) has 119,895,186, and high-coverage WGS of HGDP (referred to here as Bergstrom HGDP based on the publication) has 75,310,370. As reported previously, gnomAD has 644,267,978 high-quality SNVs and indels^17^. Because gnomAD now contains both HGDP and 1kGP, we built a synthetic subset of gnomAD that removes allele counts contributed by HGDP and 1kGP. When comparing the HGDP+1kGP dataset to this synthetic version of gnomAD that excludes HGDP+1kGP, we show that variants unique to gnomAD are disproportionately rare (Figure 4). In contrast, compared to the comprising datasets of HGDP only, the NYGC 1kGP only, and phase 3 1kGP, the HGDP+1kGP dataset uniquely contributes a sizable fraction and number of variants spanning the full allele frequency spectrum, including both rare and common variants (Figure 4). However, rare variants are particularly enriched; in all of the comparison datasets aside from gnomAD, the HGDP+1kGP dataset contains the largest proportion of rare variants. Few variants in the phase3 1kGP dataset were not in the HGDP+1kGP dataset or NYGC 1kGP because samples are entirely overlapping, as reported previously ^7^.

### Facilitating broad uptake of HGDP+1kGP as a public resource via development of detailed tutorials

In an effort to increase accessibility of this dataset, we have made publicly available tutorials of our analyses implemented primarily in Hail (https://hail.is/). Hail is an open source, Python-based, scalable tool for genomics that enables large-scale genetic analyses on the cloud. Tutorials can be accessed through Github via iPython notebooks (https://github.com/atgu/hgdp_tgp/tree/master/tutorials), and all underlying datasets are publicly available in requester-pays Google Cloud Platform buckets.

**Figure 5 |:**
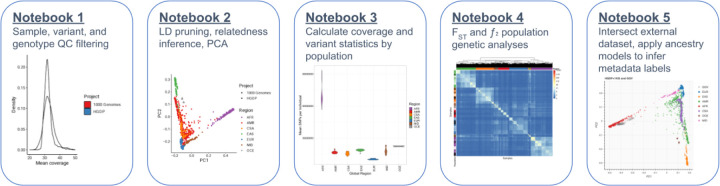
Overview of tutorials that use cloud computing to conduct common genetic data analyses. We have developed five iPython notebooks with tutorials for conducting many of the most common genetic analyses, including QC of sequencing data, relatedness inference and PCA, calculating statistics by population, analyzing genetic divergence, and applying ancestry analysis to a new dataset using HGDP+1kGP as a reference panel.

These tutorials cover various aspects of quality control (QC) and analysis, including sample and variant QC; visualizing distributions of QC statistics by metadata labels across diverse populations; filtering variants using LD, allele frequency, and missingness information; inferring relatedness; running PCA to infer ancestry; computing descriptive statistics including variant counts and coverage metrics; conducting population genetic analyses; and intersecting external datasets with HGDP+1kGP as a reference panel to apply ancestry models and infer metadata labels (Figure 5). For example, we intersected the publicly available Gambian Genome Variation (GGV) Project sequenced to low coverage with the HGDP+1kGP resource, trained a random forest on HGDP+1kGP geographical/genetic region meta-data labels, then applied this model to the GGV data to determine ancestry labels, which were all inferred to be AFR (Figure S10). When intersecting external datasets to apply ancestry labels, an important consideration is how many variants must overlap and how much missingness is tolerated to project external samples into the same PCA space as the reference panel and assign metadata labels given PCA shrinkage ^23^. We find that < 5% missingness is typically required to accurately assign ancestry labels (Figure S12 and Table S7). In addition to all these analyses, we anticipate that there will be additional uses of this resource not documented in these tutorials, such as for phasing and imputation. To facilitate these uses, we have phased the HGDP+1kGP dataset and released these phased haplotypes that others can use to support phasing and imputation in their own datasets. We have also developed computational pipelines implemented in GWASpy that use these phased reference haplotypes, and tested these tools by applying phasing and imputation to diverse samples genotyped as part of other ongoing work.

## Discussion

The 1000 Genomes Project and Human Genome Diversity Project were landmark efforts to increase the unrestricted public availability of genomic data from a geographically and ancestrally diverse set of individuals. These resources have been widely used across research efforts for decades, including as reference panels for ancestry inference, phasing, imputation, genotype refinement, and investigations into population history and demography. However, these datasets have historically been discrete, leading to suboptimal intersections when a combined analysis of all samples is required.

The harmonized variant processing, quality control, and improved coverage of variants across the allele frequency spectrum in this jointly called resource will facilitate the improved study of diverse populations. Due to our rapid release of the data pre-publication, the callset formally released here has already been used as a resource of global diversity in the Genome Aggregation Database (gnomAD) ^17^, the Pan-UK Biobank Project ^24^, the Global Biobank Meta-analysis Initiative (GBMI) ^25^, and the Covid-19 Host Genetics Initiative ^26^. A primary use of this data is as a global reference for principal components analysis (PCA)--SNV loadings are freely shared so that user cohorts can be aligned to the same PC space as this optimized reference panel. In GBMI, harmonizing ancestry analysis with this resource served as a quality control measure to ensure that ancestral groupings are being applied consistently and that control for population stratification is being performed adequately ^25^. Building on this approach and given the critical need for greater diversity in genomic studies, sequencing centers can use this resource in variant calling production pipelines to build dashboards that continuously monitor the diversity of samples being sequenced in real time.

This callset is also phased for use as a haplotype resource, potentially providing higher phasing and imputation accuracy particularly for underrepresented populations. While resources such as the Haplotype Reference Consortium (HRC) and TOPMed Imputation Panel are already helpful ^27,28^, they either provide individual-level data but lack diversity (HRC) or are very large with significant diversity but do not share individual-level data (TOPMed). This limits the application of new methods, such as those needed to support low-coverage sequencing, which is receiving growing interest as it is comparable in cost to many genotype arrays and is especially beneficial to underrepresented populations ^29^. Combinations of high-coverage exome and low-coverage genome sequencing are also of growing interest and could be uniquely supported by this resource. This resource will also be critical for developing computational and analytical tools for genotype refinement, imputation, conducting data QC particularly across varying depths of coverage, and evaluating technical biases. For example, we observed fewer SVs in the HGDP genomes than 1kGP genomes among similar ancestry groups, which was primarily explained by PCR+ and PCR-free sequencing libraries.

This resource also provides a more complete and granular capture of the full spectrum of variation across the world that would be missed by intersecting the component datasets. Because a variant’s frequency is one of its most informative features of its deleteriousness, the globally diverse allele frequencies that we have released on the gnomAD browser ^14^ provides additional scientific benefits by facilitating clinical variant interpretation across diverse populations. This GRCh38 release of this resource along with detailed tutorials for many of the most common genomic data analyses will also reduce barriers acknowledged by the vast majority of clinical labs which have not yet migrated to the latest genome build, citing that they do not feel the benefits outweigh the time and monetary costs and/or lack sufficient personnel to do so ^30^.

While this resource is more globally representative than many existing public datasets, certain geographic areas and ancestries are still underrepresented; for example, most genomic resources are enriched for participants in high-income countries ^31^ and there is particularly sparse coverage in central and southern Africa where genetic diversity is among the highest in the world. Some efforts that are already significantly underway, such as the H3Africa Initiative, will be critical for increasing representation from some of these ancestries. Other ongoing massive-scale efforts such as *All of Us* are also increasing representation from minority populations in the United States in genomics research.

As genetically diverse datasets continue to grow to massive scales, it will be invaluable for researchers to be equipped with tools and resources that facilitate scalable and efficient analysis. In the service of this goal, we concurrently release a series of detailed tutorials designed to be easily accessible in iPython notebooks demonstrating many common genomic analytic techniques as implemented in the cloud-native Hail software framework, which allows for flexible, computationally efficient, and parallelized analysis of big data. These tutorials lower the barrier for adoption of this resource and provide a code bank for researchers to conduct a variety of analyses, including conducting quality control of whole genome sequencing data, calculating variant and sample statistics within groups, analyzing population genetic variation, and applying ancestry labels from a reference panel to their own data. Overall, resources like this are essential for empowering genetic studies in diverse populations.

## Methods

### Genetic datasets

#### Human Genome Diversity Project (HGDP)

HGDP genomes sequenced and described previously ^1^ were downloaded from http://ftp.1000genomes.ebi.ac.uk/vol1/ftp/data_collections/HGDP/. Because the publicly available gVCFs were not the output of GATK HaplotypeCaller and were incompatible with joint calling, we reprocessed these genomes and conducted joint variant calling as part of gnomAD v3 ^17^. Most HGDP genomes were PCR-free (N=760), but some included PCR prior to sequencing (N=161). They were also sequenced at different times, for example as part of the Simons Genome Diversity Project (SGDP, N=120) or later at the Sanger Institute (N=801). More details are available from previous studies ^1,32^.

#### 1000 Genomes Project (1kGP)

1kGP genomes have been sequenced as part of multiple efforts, first to mid-coverage as phase 3 of the 1kGP ^4^ and more recently to high-coverage (≥30X) at the New York Genome Center (NYGC) ^7^. We used the phase 3 1kGP genomes only for comparison to previous releases. The high-coverage 1kGP genomes sequenced at the NYGC were downloaded from ftp://ftp.1000genomes.ebi.ac.uk/vol1/ftp/data_collections/1000G_2504_high_coverage/working/20201028_3202_raw_GT_with_annot/, which were harmonized with HGDP genomes to generate the HGDP+1kGP call set.

#### Human Genome Structural Variation Consortium (HGSVC)

The HGSVC generated high-coverage long-read WGS data and genomic variant calls from 34 samples in the 1kGP project (Ebert et al. 2021). We have evaluated precision of the SV callset by comparing against the long-read SV calls using these 34 genomes. The long-read SV calls were collected from (will add 1kGP ftp here)

#### Genome Aggregation Database (gnomAD)

We compared the HGDP+1kGP resource to gnomAD v3.1.2, which includes both HGDP and 1kGP high-coverage whole genomes, to quantify the extent of novel variation across the allele frequency spectrum contributed by these genomes. To generate allele counts and numbers in gnomAD that would be consistent with a fully non-overlapping set of genomes, we subtracted allele counts and allele numbers in the gnomAD variant call set that were contributed specifically by the 1kGP and HGDP genomes, effectively creating a synthetic version of gnomAD without these genomes.

#### Gambian Genome Variation Project (GGVP)

As part of tutorials that demonstrate how we can intersect an external dataset with HGDP+1kGP and assign metadata labels, we intersected the HGDP+1kGP genomes with 394 Gambian Genome Variation Project genomes which are publicly available through the IGSR (ftp://ftp.1000genomes.ebi.ac.uk/vol1/ftp/data_collections/gambian_genome_variation_project/data), as described previously ^33^. Briefly, we first downloaded GGVP CRAM files. We then used GATK HaplotypeCaller to run variant calling in GVCF mode on the 394 Gambian genomes BAM files and generated per-sample gVCFs. The single-sample gVCFs were then combined into a multi-sample Hail Sparse MatrixTable (MT) using Hail’s run_combiner() function. The GGV Sparse MT was then combined using Hail’s vcf_combiner, with the HGDP+1kGP Sparse MT to create a unique Sparse MT. Note that the Hail Sparse MatrixTable has since been replaced by the Hail VariantDataset.

### Dataset Comparisons

All of the comparison datasets used GRCh38 as their reference genome aside from phase 3 1kGP, which was on hg19 prior to liftover. The comparison datasets consisted of phase 3 of 1kGP ^4^, gnomAD v3.1.2 ^17^, high coverage HGDP whole genome sequences ^1^, and the New York Genome Center (NYGC) 1000 Genomes Project ^7^. All of these datasets were sequenced to high coverage (30X+) aside from the phase 3 1000 Genomes Project, which was sequenced to 4–8X coverage. The NYGC dataset includes all of the original 2,504 samples from phase3 1kGP as well as an additional 698 related samples.

### Sample and variant QC

Quality control of samples was conducted according to procedures used in gnomAD ^34^ but was then modified to relax some gnomAD sample QC filters new to v3 in especially diverse or unique genomes. Specifically, the filters starting with ‘fail_’ indicate whether samples are outliers in number of variants after regressing out principal components, which can indicate a sample issue. However, we identified whole continental groups and populations that were removed due solely to SNV and indel residual filters, especially those that were most genetically unique (i.e., San, Mbuti Pygmy, Biaka Pygmy, Bougainville, and Papuan). Additional individuals from the LWK, Bantu Kenya, and Bantu South Africa populations were also removed solely on the basis of the fail_n_snp_residual filter.

Before running any quality control filters, there were 211,358,784 variants and 4,151 samples. We then applied gnomAD sample QC filters (excluding the filters starting with ‘fail_’), which removed 31 samples. Next, we identified 22 ancestry outliers by conducting global and subcontinental PCA within metadata genetic region labels (AFR, AMR, CSA, EAS, EUR, MID, and OCE), and removed individuals who deviated substantially in PC space from others with the same metadata label along the first 10 PCs. We also removed a duplicate sample. Lastly, we subset to only variants which were flagged as passing the gnomAD QC pipeline, as described previously ^34^. After filtering, there were 155,648,020 variants and 4,096 individuals included in the dataset.

The number of SNVs was calculated using Hail’s sample_qc() method. Because singletons are especially sensitive to variation in sample size per population which is substantial across HGDP and 1kGP, we compared singleton counts by randomly downsampling to 6 unrelated samples, the minimum number of individuals per population, then removed monomorphic variants. Coverage data was computed in gnomAD from the bam metrics field. We then calculated the mean of these metrics per individual within a population using Hail’s hl.agg.stats() method ^35^.

### Relatedness

We computed relatedness using the PC-Relate algorithm ^36^ implemented in Hail. Specifically, we considered SNVs with a minor allele frequency of 0.05, 20 PCs, and allowed kinship coefficients up to 0.05 using the min_individual_maf=0.05, min_kinship=0.05, statistics=‘kin’, k=20 arguments.

### PCA

We computed 20 PCs across global populations as well as within each continental ancestry group according to the Genetic.region project metadata harmonized across HGDP and 1kGP as shown in Table S1. We first filtered to samples and variants that passed QC. We required that SNVs have MAF > 0.05 and missingness < 0.1%. We then performed LD pruning within a 500kb window, restricting to variants with r^2^ < 0.1, leaving 255,666 variants for analysis. Finally, we computed relatedness as described above and restricted to a maximally independent set of unrelated individuals.

Using this filtered dataset, we ran PCA both globally and within metadata labels (AFR, AMR, CSA, EAS, EUR, MID, and OCE) in unrelated individuals using Hail’s hwe_normalized_pca() function, then projected related individuals into that PC space using a pc_project() function used in gnomAD and implemented in Hail.

### Structural variants

#### Initial SV discovery and pruning

We applied GATK-SV ^18^ to integrate and genotype SVs from the HGDP and 1kGP samples. Briefly, the HGDP samples were split into batches, each consisting of ~190 samples, based on their initial cohort, PCR status, sex, and sequencing depth of the libraries (Figure S3). Raw Initial SVs were detected per sample by Manta ^37^, Wham ^38^, cnMOPs ^39^, and GATK-gCNV ^40^ and then were clustered across each batch and filtered through an initial random forest machine learning model to remove potential false positive SVs. We then jointly genotyped SVs across all batches using a non-redundant union of SVs. Partially overlapping SVs were either re-clustered into a unique SV or resolved into complex events. We observed mosaicism resulting from gain or loss of X and Y chromosomes for several samples (Table S5), likely due to a cell line artifact from passaging. While mosaic loss of the Y chromosome is the most common form of clonal mosaicism ^41^, the non-canonical sex chromosome ploidies observed are not unique to these samples and have been previously observed in other datasets ^7,18^.

#### SV refinement and annotation

A series of refinements have been applied to improve the precision of SV calls while maintaining high sensitivity. First, two machine learning models have been developed and applied to prune false positive SVs. A lightGBM model has been trained on the 9 1kGP samples that have been deep sequenced with long-read WGS data by the HGSVC ^20,42^, and applied to all SVs except for large bi-allelic CNVs (>5Kb). Details of the lightGBM model can be found in ^7^. Meanwhile, a minGQ model has been trained using the inheritance information among trio families to filter bi-allelic CNVs that are 5Kb and above. Details of this model can be found in ^18^. Genomes that failed the machine learning models were assigned a null genotype, and the proportion of null genotypes among all samples were calculated as an “no call rate” (NCR) score. SV sites that have a 10% or higher NCR were labeled as low quality variants and removed from further analyses. Then, we examined the distribution of SVs per genome to identify potential outlier samples that carry significantly more SVs than average, and also compared the frequency of SVs across each batch to identify SVs that showed significant bias (i.e. batch effects). The resulting SV callset were annotated with their frequency by their ancestry.

#### Phased haplotypes

The haplotype phasing was split into three steps: (1) scatter; (2) phasing; (3) concatenation. Firstly, the data was split into chunks of window size 10.0cM with 2.0cM overlap between each chunk. SHAPEIT4 was then used to phase each chunk. The phased chunks were then concatenated into chromosomes using bcftools. For the chunk in the *chr1:120246522-149637342* region, SHAPEIT4 always failed after the second main iteration step of MCMC when the default *5b,1p,1b,1p,1b,1p,5m* sequence was used. As a workaround, we lowered the number of main iterations in the chunk to 2 from 5 (*5b,1p,1b,1p,1b,1p,2m*).

## Data availability

All data are freely available and described more completely here: https://gnomad.broadinstitute.org/news/2020-10-gnomad-v3-1-new-content-methods-annotations-and-data-availability/#the-gnomad-hgdp-and-1000-genomes-callset. Phased haplotypes are available in BCFs here: gs://gcp-public-data--gnomad/resources/hgdp_1kg/phased_haplotypes/.

## Supplementary Material

Supplement 1

Supplement 2

## References

[1] Bergström A. Insights into human genetic variation and population history from 929 diverse genomes. Science 367, (2020). 10.1126/science.aay5012 PMC7115999 32193295

[2] Rosenberg N. A. Genetic structure of human populations. Science 298, 2381–2385 (2002). 12493913 10.1126/science.1078311

[3] Li J. Z. Worldwide human relationships inferred from genome-wide patterns of variation. Science 319, 1100–1104 (2008). 18292342 10.1126/science.1153717

[4] 1000 Genomes Project Consortium A global reference for human genetic variation. Nature 526, 68–74 (2015). 26432245 10.1038/nature15393 PMC4750478

[5] 1000 Genomes Project Consortium An integrated map of genetic variation from 1,092 human genomes. Nature 491, 56–65 (2012). 23128226 10.1038/nature11632 PMC3498066

[6] Ebert P., Audano P. A., Zhu Q. & Rodriguez-Martin B. De novo assembly of 64 haplotype-resolved human genomes of diverse ancestry and integrated analysis of structural variation. bioRxiv (2020).

[7] Byrska-Bishop M. High-coverage whole-genome sequencing of the expanded 1000 Genomes Project cohort including 602 trios. Cell 185, 3426–3440.e19 (2022). 36055201 10.1016/j.cell.2022.08.004 PMC9439720

[8] Lam M. RICOPILI: Rapid Imputation for COnsortias PIpeLIne. Bioinformatics 36, 930–933 (2020). 31393554 10.1093/bioinformatics/btz633 PMC7868045

[9] Howie B., Fuchsberger C., Stephens M., Marchini J. & Abecasis G. R. Fast and accurate genotype imputation in genome-wide association studies through pre-phasing. Nat. Genet. 44, 955–959 (2012). 22820512 10.1038/ng.2354 PMC3696580

[10] Cavalli-Sforza L. L., Wilson A. C., Cantor C. R., Cook-Deegan R. M. & King M. C. Call for a worldwide survey of human genetic diversity: a vanishing opportunity for the Human Genome Project. Genomics 11, 490–491 (1991). 1769670 10.1016/0888-7543(91)90169-f

[11] Cavalli-Sforza L. L. The Human Genome Diversity Project: past, present and future. Nat. Rev. Genet. 6, 333–340 (2005). 15803201 10.1038/nrg1596

[12] Karczewski K. J. The mutational constraint spectrum quantified from variation in 141,456 humans. Nature 581, 434–443 (2020). 32461654 10.1038/s41586-020-2308-7 PMC7334197

[13] Taliun D. Sequencing of 53,831 diverse genomes from the NHLBI TOPMed Program. Nature 590, 290–299 (2021). 33568819 10.1038/s41586-021-03205-y PMC7875770

[14] Karczewski K. J. The ExAC browser: displaying reference data information from over 60 000 exomes. Nucleic Acids Res. 45, D840–D845 (2017). 27899611 10.1093/nar/gkw971 PMC5210650

[15] Maples B. K., Gravel S., Kenny E. E. & Bustamante C. D. RFMix: a discriminative modeling approach for rapid and robust local-ancestry inference. Am. J. Hum. Genet. 93, 278–288 (2013). 23910464 10.1016/j.ajhg.2013.06.020 PMC3738819

[16] Rubinacci S., Ribeiro D. M., Hofmeister R. J. & Delaneau O. Efficient phasing and imputation of low-coverage sequencing data using large reference panels. Nat. Genet. 53, 120–126 (2021). 33414550 10.1038/s41588-020-00756-0

[17] Chen S. A genome-wide mutational constraint map quantified from variation in 76,156 human genomes. bioRxiv 2022.03.20.485034 (2022) doi:10.1101/2022.03.20.485034.

[18] Collins R. L. A structural variation reference for medical and population genetics. Nature 581, 444–451 (2020). 32461652 10.1038/s41586-020-2287-8 PMC7334194

[19] Sudmant P. H. An integrated map of structural variation in 2,504 human genomes. Nature 526, 75–81 (2015). 26432246 10.1038/nature15394 PMC4617611

[20] Ebert P. Haplotype-resolved diverse human genomes and integrated analysis of structural variation. Science 372, (2021). 10.1126/science.abf7117 PMC8026704 33632895

[21] Marcus J. H. & Novembre J. Visualizing the geography of genetic variants. Bioinformatics 33, 594–595 (2017). 27742697 10.1093/bioinformatics/btw643 PMC5408806

[22] Ramachandran S. Support from the relationship of genetic and geographic distance in human populations for a serial founder effect originating in Africa. Proc. Natl. Acad. Sci. U. S. A. 102, 15942–15947 (2005). 16243969 10.1073/pnas.0507611102 PMC1276087

[23] Dey R. & Lee S. Asymptotic properties of principal component analysis and shrinkage-bias adjustment under the generalized spiked population model. J. Multivar. Anal. 173, 145–164 (2019). 32831421 10.1016/j.jmva.2019.02.007 PMC7441582

[24] Karczewski K. Pan-UK Biobank. https://pan.ukbb.broadinstitute.org/.

[25] Zhou W. Global Biobank Meta-analysis Initiative: Powering genetic discovery across human disease. Cell Genomics 2, (2022). 10.1016/j.xgen.2022.100192 PMC9903716 36777996

[26] The COVID-19 Host Genetics Initiative & Ganna, A. Mapping the human genetic architecture of COVID-19 by worldwide meta-analysis. bioRxiv (2021) doi:10.1101/2021.03.10.21252820.

[27] McCarthy S. A reference panel of 64,976 haplotypes for genotype imputation. Nat. Genet. 48, 1279–1283 (2016). 27548312 10.1038/ng.3643 PMC5388176

[28] Kowalski M. H. Use of >100,000 NHLBI Trans-Omics for Precision Medicine (TOPMed) Consortium whole genome sequences improves imputation quality and detection of rare variant associations in admixed African and Hispanic/Latino populations. PLoS Genet. 15, e1008500 (2019). 31869403 10.1371/journal.pgen.1008500 PMC6953885

[29] Martin A. R. Low-coverage sequencing cost-effectively detects known and novel variation in underrepresented populations. Am. J. Hum. Genet. 108, 656–668 (2021). 33770507 10.1016/j.ajhg.2021.03.012 PMC8059370

[30] Lansdon L. A. Factors affecting migration to GRCh38 in laboratories performing clinical next-generation sequencing. J. Mol. Diagn. (2021) doi:10.1016/j.jmoldx.2021.02.003. 33631350

[31] Fatumo S. A roadmap to increase diversity in genomic studies. Nat. Med. 28, 243–250 (2022). 35145307 10.1038/s41591-021-01672-4 PMC7614889

[32] Mallick S. The Simons Genome Diversity Project: 300 genomes from 142 diverse populations. Nature 538, 201–206 (2016). 27654912 10.1038/nature18964 PMC5161557

[33] Network M. G. E. & Malaria Genomic Epidemiology Network. Insights into malaria susceptibility using genome-wide data on 17,000 individuals from Africa, Asia and Oceania. Nature Communications vol. 10 Preprint at 10.1038/s41467-019-13480-z (2019). PMC6914791 31844061

[34] gnomAD v3.1 New Content, Methods, Annotations, and Data Availability. https://gnomad.broadinstitute.org/blog/2020-10-gnomad-v3-1-new-content-methods-annotations-and-data-availability/.

[35] Hail | Aggregators. https://hail.is/docs/0.2/aggregators.html.

[36] Conomos M. P., Reiner A. P., Weir B. S. & Thornton T. A. Model-free Estimation of Recent Genetic Relatedness. Am. J. Hum. Genet. 98, 127–148 (2016). 26748516 10.1016/j.ajhg.2015.11.022 PMC4716688

[37] Chen X. Manta: rapid detection of structural variants and indels for germline and cancer sequencing applications. Bioinformatics 32, 1220–1222 (2016). 26647377 10.1093/bioinformatics/btv710

[38] Kronenberg Z. N. Wham: Identifying Structural Variants of Biological Consequence. PLoS Comput. Biol. 11, e1004572 (2015). 26625158 10.1371/journal.pcbi.1004572 PMC4666669

[39] Klambauer G. cn.MOPS: mixture of Poissons for discovering copy number variations in next-generation sequencing data with a low false discovery rate. Nucleic Acids Res. 40, e69 (2012). 22302147 10.1093/nar/gks003 PMC3351174

[40] Babadi M. GATK-gCNV: A Rare Copy Number Variant Discovery Algorithm and Its Application to Exome Sequencing in the UK Biobank. bioRxiv 2022.08.25.504851 (2022) doi:10.1101/2022.08.25.504851.

[41] Thompson D. J. Genetic predisposition to mosaic Y chromosome loss in blood. Nature 575, 652–657 (2019). 31748747 10.1038/s41586-019-1765-3 PMC6887549

[42] Chaisson M. J. P. Multi-platform discovery of haplotype-resolved structural variation in human genomes. Nat. Commun. 10, 1784 (2019). 30992455 10.1038/s41467-018-08148-z PMC6467913

