# Supplementary material for: A harmonized public resource of deeply sequenced diverse human genomes
Source: bioRxiv. 2023 Aug 10:2023.01.23.525248. Preprint. [Version 3] doi: 10.1101/2023.01.23.525248 (PMC9900804; doi:10.1101/2023.01.23.525248)
Supplement: Supplementary file 1 [file media-1.pdf]

HGDP+1kGP supplement

HGDP+1kGP supplement **1**
Data harmonization 1
Genotype data 1
Metadata 1
Table S1 | Harmonization of HGDP and 1000 Genomes Project meta-data project labels. 1
Table S2 | Genetic outliers identified in analysis of global and subcontinental PCA. 1
Table S3 | Final sample counts. 1
QC Metadata Summaries 2
Figure S1 | Coverage across the 1kGP and HGDP. 2
Figure S2 | Coverage across 1kGP and HGDP by population. 2
Table S4 | Coverage as well as SNV and SV statistics by population. 2
Structural variants (SVs) 2
Figure S3 | Dosage and sex ploidy of HGDP samples and batching strategy. 2
Figure S4 | SV callset and quality evaluation results. 2
Table S5 | Sex chromosome aneuploidies in the HGDP samples. 2
Figure S5 | Mean count of SVs versus SNVs by project, region, and number of individuals. 2
Table S6 | SV calls by external support from HGSV study. 3
Figure S6 | SV breakdown in count by class across HGDP and 1kGP (HGSV). 3
Population genetic comparisons 3
Figure S7 | ADMIXTURE analysis of the HGDP and 1kGP resource. 3
Figure S8 | 5-fold cross-validation error across ADMIXTURE runs. 3
Figure S9 | PCA biplots and densities globally. 4
Figure S10 | Subcontinental PCA in AFR populations. 4
Figure S11 | Subcontinental PCA in CSA populations. 5
Figure S12 | Subcontinental PCA in EAS populations. 5
Figure S13 | Subcontinental PCA in EUR populations. 5
Figure S14 | Subcontinental PCA in AMR populations. 6
Figure S15 | Subcontinental PCA in MID populations. 6
Figure S16 | Subcontinental PCA in OCE populations. 6
Figure S17 | HGDP+1kGP ancestry labels applied to the Gambian Genome Variation (GGV) Project. 7
Figure S18 | Dendrogram of the pairwise FST heatmap between populations colored by geographical/genetic regions. 7
Table S7 | Populations interspersed among other geographical/genetic regions obtained from the pairwise FST heatmap, colored by region. 8
Table S8 | Pearson's correlation and Mantel tests results with and without waypoints. 11
Quality control 12
Figure S19 | Example of a filter that was included in gnomAD v3.1 but excluded from this project. 13
Analysis tutorials 13
Figure S20 | PCA shrinkage analysis to determine acceptable levels of missingness before ancestry resolution becomes too low to accurately assign population labels. 14

Table S9 | Shrinkage analysis matches and no classification numbers by SNP missingness in the test dataset, as shown in Figure S20. 16
References 16

Data harmonization

Genotype data

Genotype data was processed as described in [1]. Briefly, reads were mapped using BWA-MEM, cleaned using the GATK Best Practices pipeline, and gVCFs were generated using GATK HaplotypeCaller. Joint calling was performed using the Hail combiner [2] and converted to a VariantDataset (VDS), which was then densified into a dense MatrixTable used for analysis. These datasets are released on Google Cloud Platform, Amazon Web Services, and Microsoft Azure, and can be found on the Downloads page of the gnomAD browser (https://gnomad.broadinstitute.org/downloads#v3-hgdp-1kg).

Metadata

Where possible, we combined meta-data from the 1000 Genomes Project and HGDP by combining the "super population" data from the 1000 Genomes project [3] and region information from HGDP [4]. We created a harmonized combined label with 3-letter codes for all groups, which we refer to as geographical/genetic region throughout the text. Where a region was only clearly contained in HGDP, we used the HGDP information to define a 3-letter code. The CENTRAL_SOUTH_ASIA code contained within HGDP is more geographically expansive than the SAS label contained in the 1000 Genomes Project, so we expanded the 3 letter code to be CSA, as shown in **Table S1**.

Table S1 | Harmonization of HGDP and 1000 Genomes Project meta-data project labels.
These labels are referred to as geographical/genetic regions throughout this manuscript. The sample sizes included here are pre-QC. Post-QC numbers are shown in **Table S3**.

1000 Genomes super population	HGDP region	Combined label (geographical/ genetic region)	Sample size (pre-QC)
AFR	AFRICA	AFR	1003
AMR	AMERICA	AMR	552
SAS	CENTRAL_SOUTH_ASIA	CSA	790
EAS	EAST_ASIA	EAS	825
EUR	EUROPE	EUR	788
N/A	MIDDLE_EAST	MID	162
N/A	OCEANIA	OCE	30

After combining region data, we used principal components analysis (PCA) to identify ancestry outliers within regions. We identified outliers as described in **Table S2** and provide final sample counts in **Table S3**.

Table S2 | Genetic outliers identified in analysis of global and subcontinental PCA.
Within subcontinental PCA biplots, outliers were identified when one to three samples defined most of an entire PC among PCs 1-6.

Sample ID	Region	Population
HG01880	AFR	ACB
HG01881	AFR	ACB
NA20274	AFR	ASW
NA20299	AFR	ASW
NA20314	AFR	ASW
HGDP00013	CSA	Brahui
HGDP00029	CSA	Brahui
HGDP00057	CSA	Balochi
HGDP00130	CSA	Makrani
HGDP00150	CSA	Makrani
HGDP00175	CSA	Sindhi
HGDP01298	EAS	Uygur
HGDP01300	EAS	Uygur
HGDP01303	EAS	Uygur
LP6005443-DNA_B02	EAS	Uygur
HG01628	EUR	IBS
HG01629	EUR	IBS
HG01630	EUR	IBS
HG01694	EUR	IBS
HG01696	EUR	IBS
HGDP00621	MID	Bedouin
HGDP01270	MID	Mozabite
HGDP01271	MID	Mozabite
CHMI_CHMI3_WGS2	gnomAD QC sample (not PCA outlier)	

Table S3 | Final sample counts.
Note: hard filtering was performed as in gnomAD v3 with modifications as described below (in the initial gnomAD release, 3,280 of these 4,120 hard filtered individuals are included). The first two rows of the Total column include a "synthetic diploid" QC sample (CHM; Complete Hydatidiform Mole) described previously[5]. It was removed with PCA outliers and excluded from sample counts reported in the manuscript.

	HGDP	1kGP	Total
Initial dataset	948	3,202	4,151
Hard filtered	943	3,176	4,120
PCA outliers removed	930	3,166	4,096
Unrelated individuals	871	2,507	3,378

QC Metadata Summaries

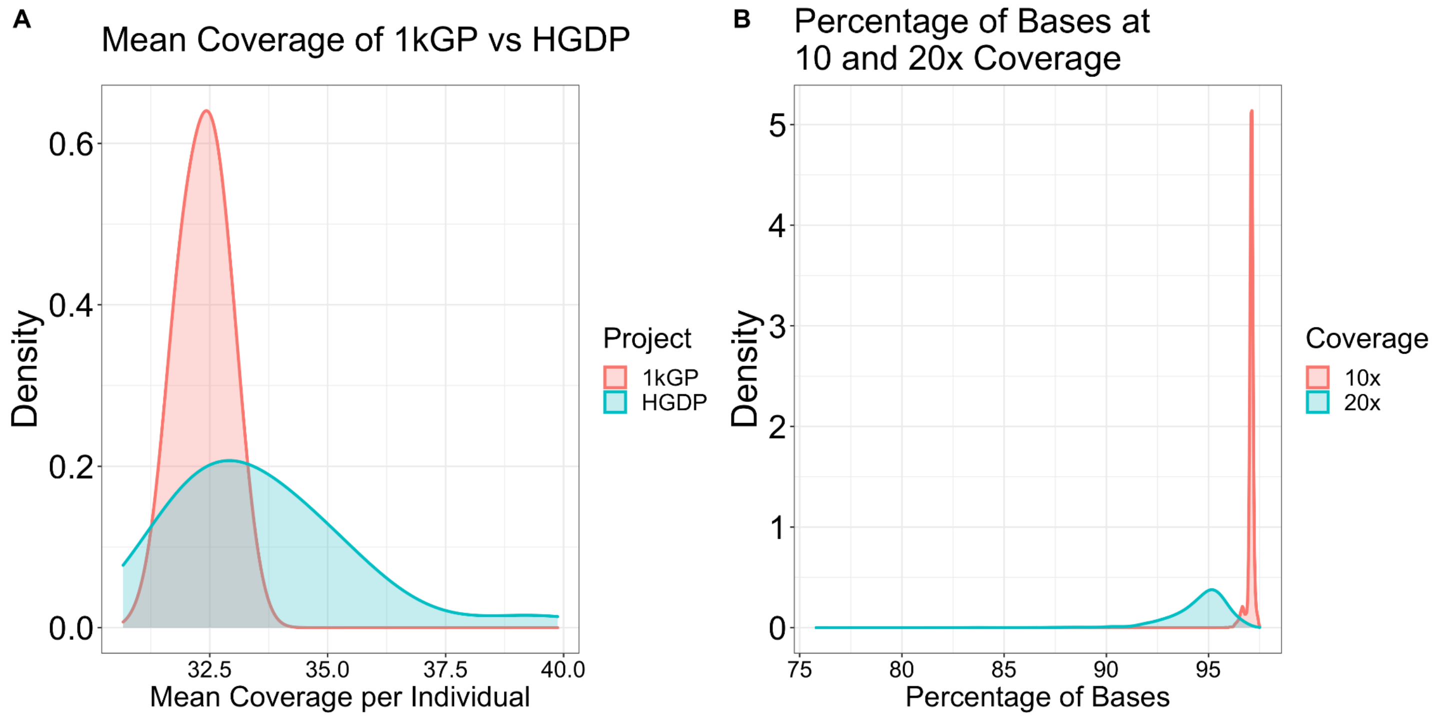

Figure S1 | Coverage across the 1kGP and HGDP.
A) Coverage in both datasets is uniformly above 30X, with an average of 33X coverage across the harmonized dataset. The coverage of the HGDP genomes is more variable than in 1kGP, as expected based on a variety of technical differences such as multiple sequencing batches, PCR+ vs PCR-free, and older cell lines in HGDP compared to 1kGP. The differences in project coverages also impacts the distribution of coverage statistics by Geographical region given their tally by project (**Table S4**). The overall coverage distributions by population are shown in **Figure S2**. B) Over 95% of bases are covered over 10X, and over 90% of bases are covered over 20X in HGDP+1kGP.

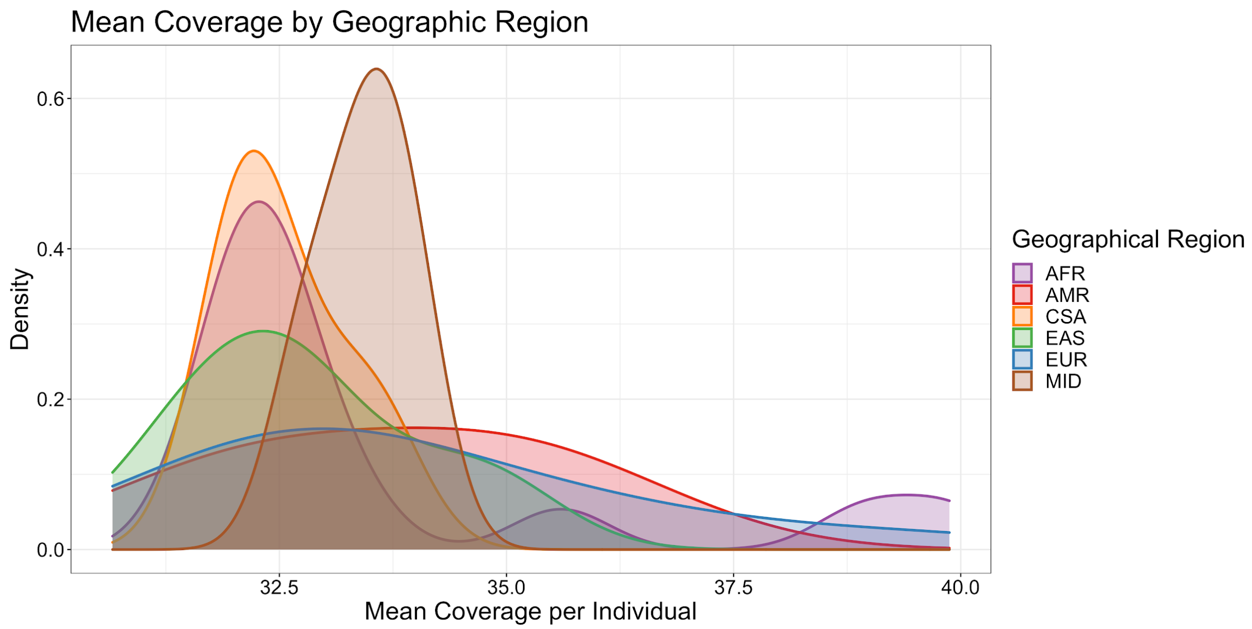

Figure S2 | Coverage across 1kGP and HGDP by population.
Regional abbreviations are as described in **Table S1**. OCE is excluded from this plot as it is represented by only two populations. Mean coverage across the different regions is 33X with coverage consistently above 30X for all regions.

Table S4 | Coverage as well as SNV and SV statistics by population.
Coverage was computed across the genome as part of the gnomAD project. Relatedness was inferred using PC-Relate. Because number of variants and singleton counts per individual are sensitive to sample size imbalances, they were tallied using a downsampled version of the dataset in which each population was randomly downsampled to match the smallest population (i.e. 6 individuals per population), then SNVs were removed if they were not polymorphic in the downsampled dataset. Given the more pronounced impact of batch effects on structure variant (SV) calling and the number of batches present within and between datasets, the number of SVs per individual were calculated across the full dataset, not in the downsampled dataset.

Structural variants (SVs)

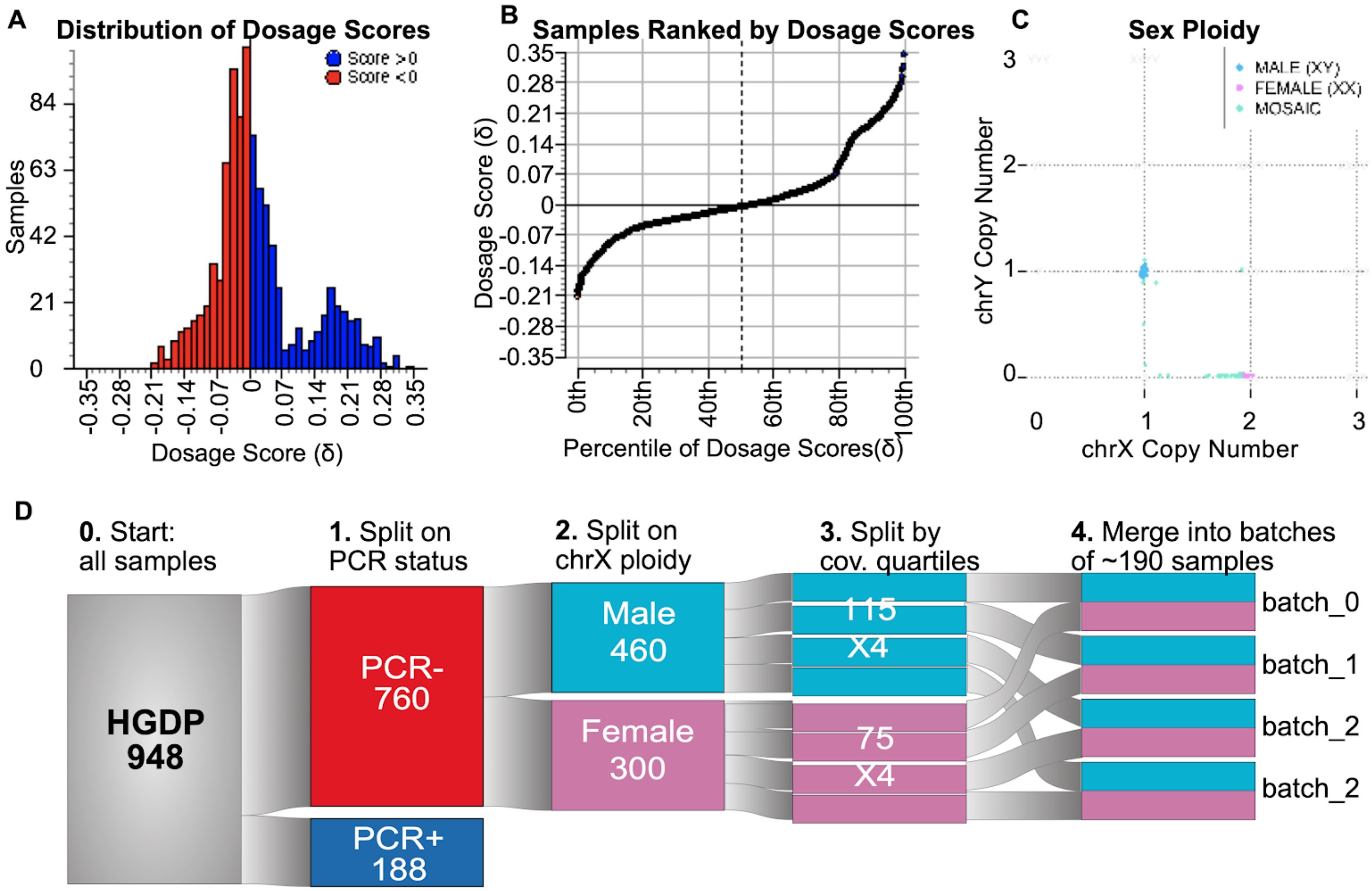

Figure S3 | Dosage and sex ploidy of HGDP samples and batching strategy.
A) Distribution of dosage scores across HGDP samples. We used the previously developed whole genome dosage model (Collins et al 2020) to quantify non-uniform distribution of sequencing coverage.The dosage scores corresponded predominantly to PCR-amplified (PCR+) and PCR-free (PCR-) library protocols. B) Samples ranked by dosage score. C) Distribution of chrX copy number across HGDP samples. D) Batching strategy for SV calling. HGDP samples were first split by their PCR status and chrX ploidy. PCR- samples were then ranked by their sequencing depth from low to high, and split into four sub batches of equivalent sizes. Male and female batches with matched coverage quantiles are combined to form the final batches.

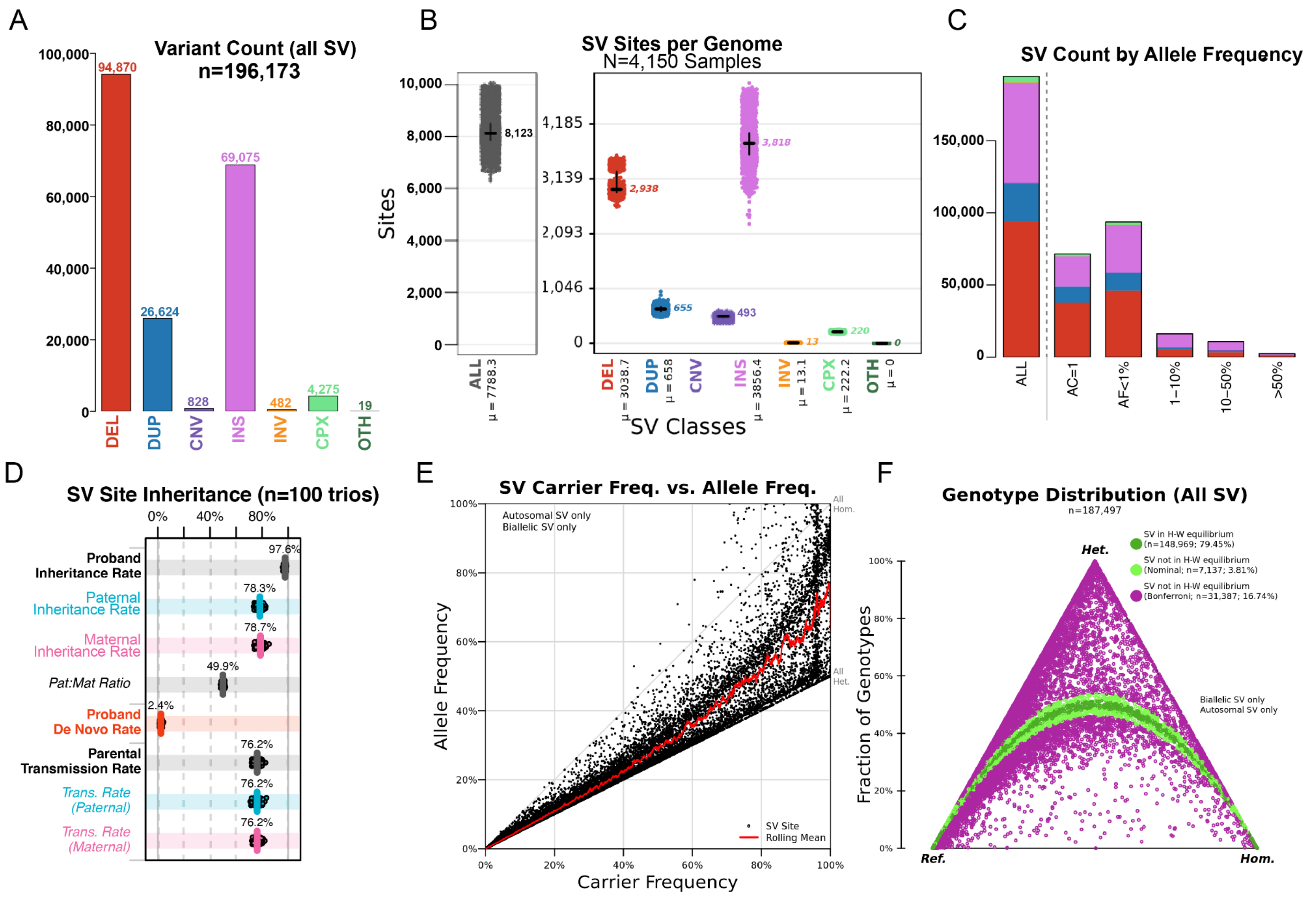

Figure S4 | SV callset and quality evaluation results.

A) Count of SV sites across 4,150 HGDP and 1kGP samples by variant type. B) Count of SVs per genome by variant type. C) Count of SV sites by allele frequency. D) Inheritance of SVs calculated in 100 pather-mother-child trio families. E) Correlation of allele frequencies. F) Hardy-Weinberg Equilibrium distribution of SVs.

Table S5 | Sex chromosome aneuploidies in the HGDP samples.

Sample ID	Population	Genetic region	chrX	chrY	Assignment
HGDP00445	Burusho	CSA	1	0	XO
HGDP01157	Bergamo Italian	EUR	1	0	XO
HGDP01208	Oroquen	EAS	2	1	XXY
HGDP01368	Basque	EUR	1	0	XO
LP6005441-DNA_G09	Palestinian	MID	1	0	XO

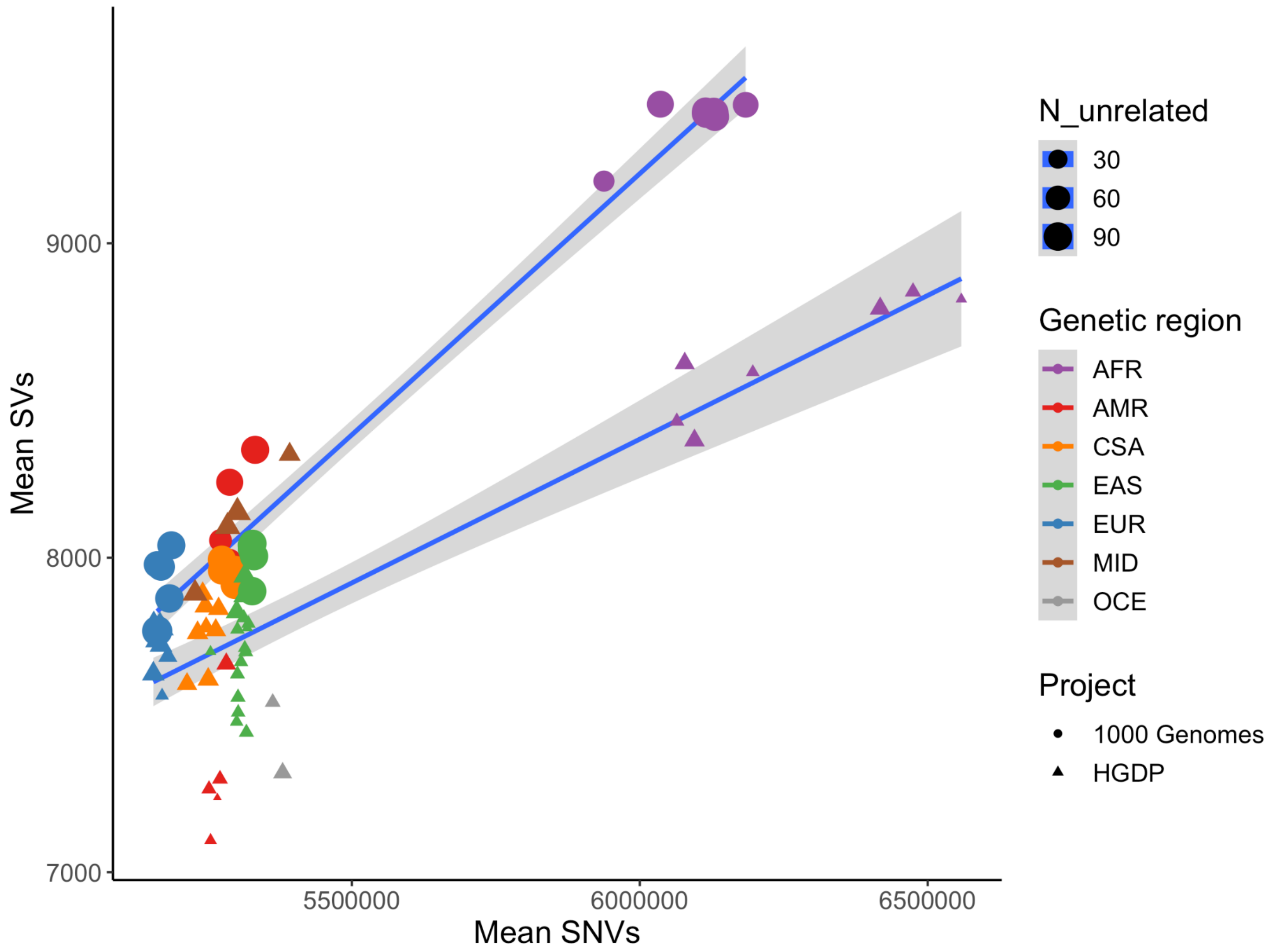

Figure S5 | Mean count of SVs versus SNVs by project, region, and number of individuals.
Top line shows a fitted regression line to the 1000 Genomes Project points, and bottom line is fitted to HGDP points. A larger number of SVs are present in the 1000 Genomes Project data, which was explored more fully in **Figure S6**.

Table S6 | SV calls by external support from HGSV study.

		External Supports (Count SVs per genome)			
SVtype	Precision	No Support	Illumina	PacBio	Illumina and PacBio
DEL	97.60%	74	116	205	2688
DUP	89.30%	73	34	216	359
INS	91.37%	346	456	320	2889
INV	85.71%	2	0	12	0
CNV	71.29%	143	12	308	35
CPX	75.89%	54	0	170	0
All-SVs	91.87%	692	618	5971	1231

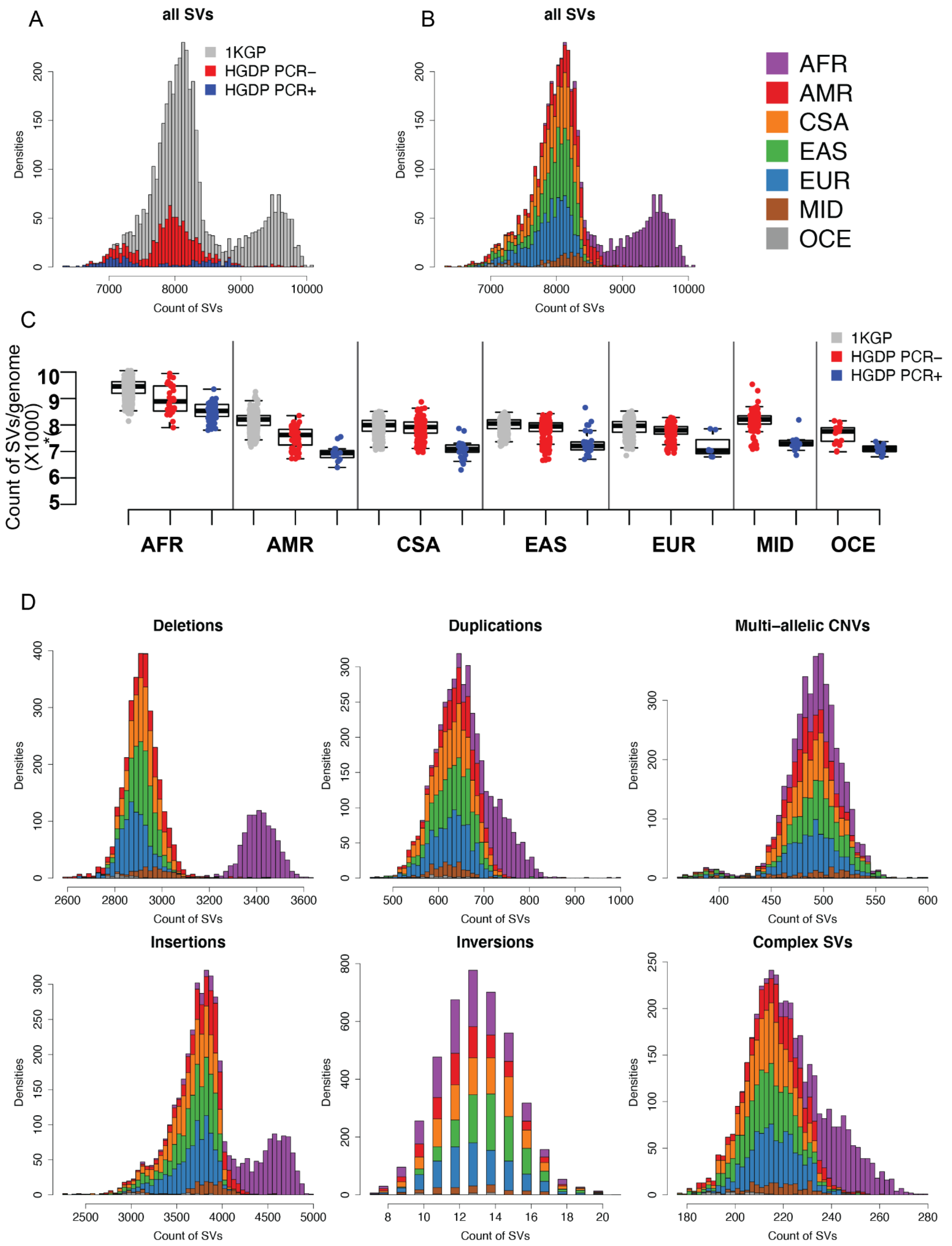

Figure S6 | SV breakdown in count by class across HGDP and 1kGP (HGSV).
Per genome SV counts by study and PCR status (A,C), and population (B). Per genome SV counts are also broken down by SV type, including deletions, duplications, multi-allelic CNVs, insertions, inversions, and complex SVs in D).

Population genetic comparisons

The breakdown of ancestry and population structure by ADMIXTURE is similar to that identified in global PCA, with K=2 highlighting structure in the AFR, K=3 highlighting structure in the EAS, K=4 highlighting structure in the EUR and CSA, K=5 highlighting structure in the AMR, K=6 highlighting structure in the OCE, K=7 highlighting structure in the MID, and subsequent values of K highlighting structure within meta-data labels (**Figure S7**).

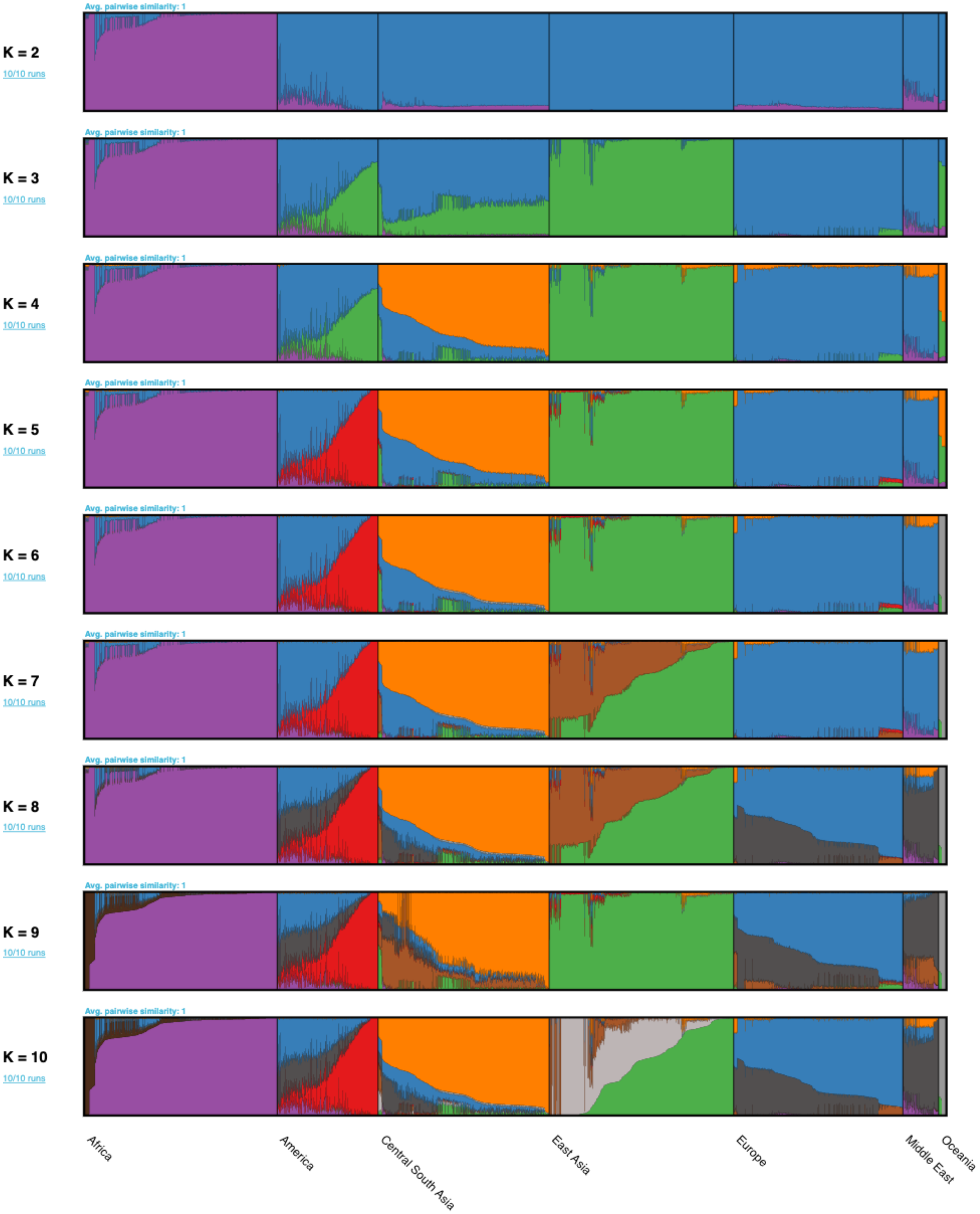

Figure S7 | ADMIXTURE analysis of the HGDP and 1kGP resource.
We ran ADMIXTURE with values of K=2 through K=10 across populations and harmonized geographical/genetic regions. Each row of bar plots shows the breakdown of regional substructure as K increases, where K is the number of genetic ancestry components fit in that run. For example, when K=2, AFR separates from the rest of the populations as the most distinct population due to high levels of genetic diversity. When K=3 EUR separates from the rest, and so on. We chose the best fit value of K to be K=6 based on a reduction in the rate of change of 5-fold cross validation error as shown in **Figure S8**.

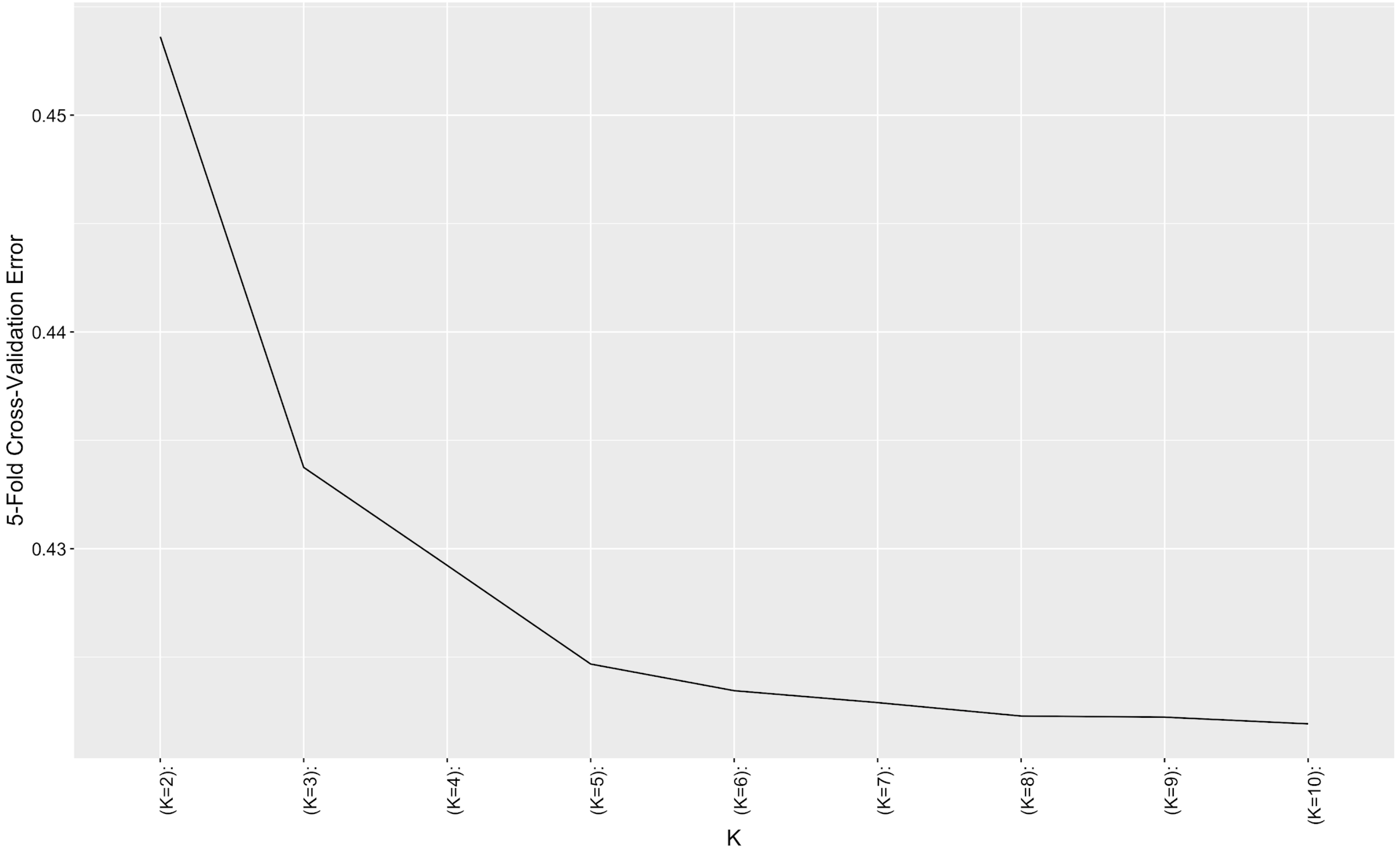

Figure S8 | 5-fold cross-validation error across ADMIXTURE runs.
We selected K=6 as the point at which cross-validation error leveled out. As described in the ADMIXTURE manual, the cross-validation error enables users to identify the value of K for which the model has best predictive accuracy, as determined by “holding out” data points. It partitions observed genotypes into 5 roughly equally sized folds, masks genotypes for each fold, then predicts the genotypes.

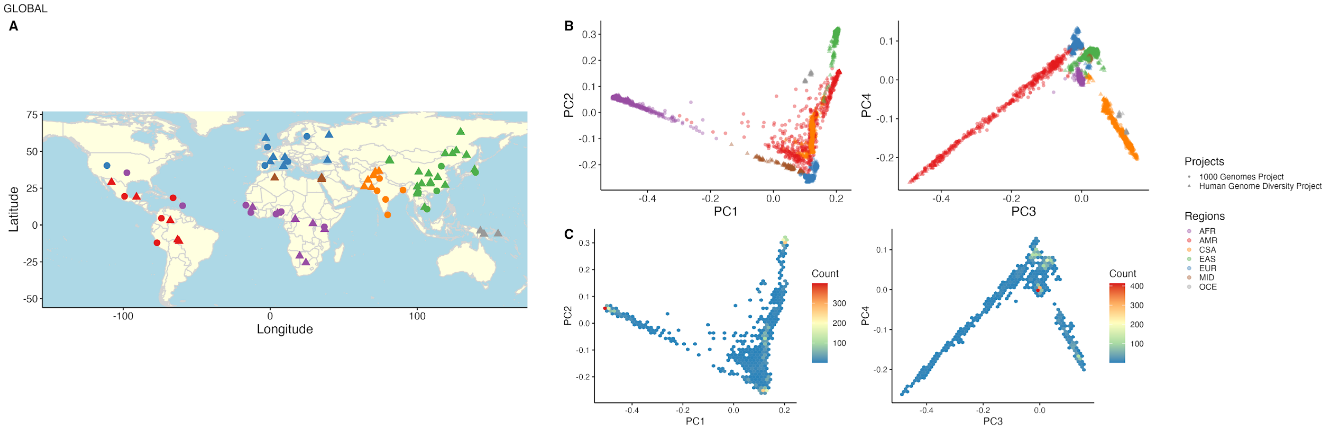

Figure S9 | PCA biplots and densities globally.
A) Map shows where all samples in analyses are from. B) PCA biplots of PCs 1-4. PCA outliers were removed prior to this analysis. Filled circles indicate populations in the 1000 Genomes Project, while filled triangles indicate populations in HGDP. Population codes are as in **Table S1**. C) Density plot of PCA biplots of PCs 1-4.

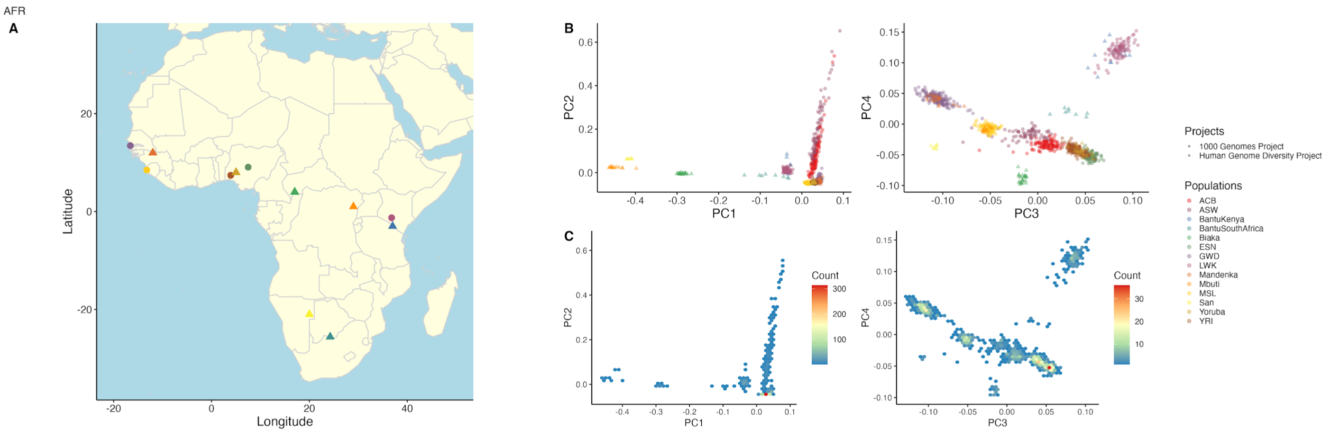

Figure S10 | Subcontinental PCA in AFR populations.
A) Map shows where all AFR samples in analyses are from. B) PCA biplots of PCs 1-4. PCA outliers were removed prior to this analysis. Filled circles indicate populations in the 1000 Genomes Project, while filled triangles indicate populations in HGDP. Population codes are as in **Table S1**. C) Density plot of PCA biplots of PCs 1-4.

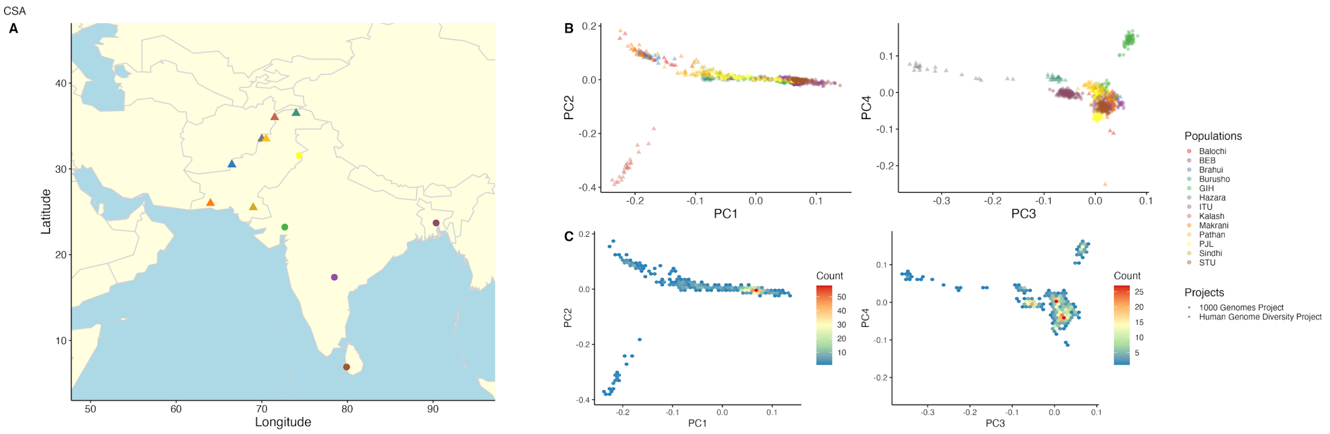

Figure S11 | Subcontinental PCA in CSA populations.
A) Map shows where all CSA samples in analyses are from. B) PCA biplots of PCs 1-4. PCA outliers were removed prior to this analysis. Filled circles indicate populations in the 1000 Genomes Project, while filled triangles indicate populations in HGDP. Population codes are as in **Table S1**. C) Density plot of PCA biplots of PCs 1-4.

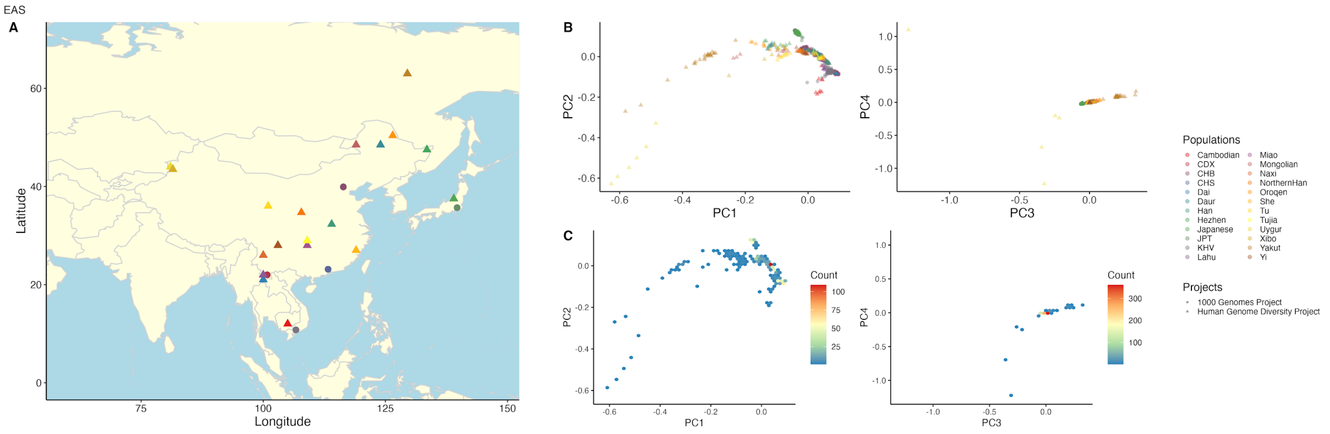

Figure S12 | Subcontinental PCA in EAS populations.
A) Map shows where all EAS samples in analyses are from. B) PCA biplots of PCs 1-4. PCA outliers were removed prior to this analysis. Filled circles indicate populations in the 1000 Genomes Project, while filled triangles indicate populations in HGDP. Population codes are as in **Table S1**. C) Density plot of PCA biplots of PCs 1-4.

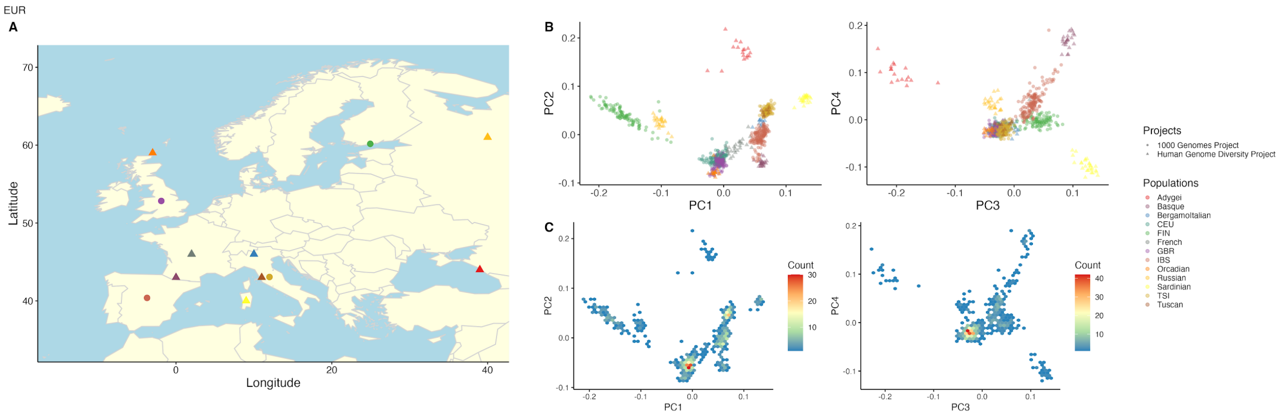

Figure S13 | Subcontinental PCA in EUR populations.
A) Map shows where all EUR samples in analyses are from. B) PCA biplots of PCs 1-4. PCA outliers were removed prior to this analysis. Filled circles indicate populations in the 1000 Genomes Project, while filled triangles indicate populations in HGDP. Population codes are as in **Table S1**. C) Density plot of PCA biplots of PCs 1-4.

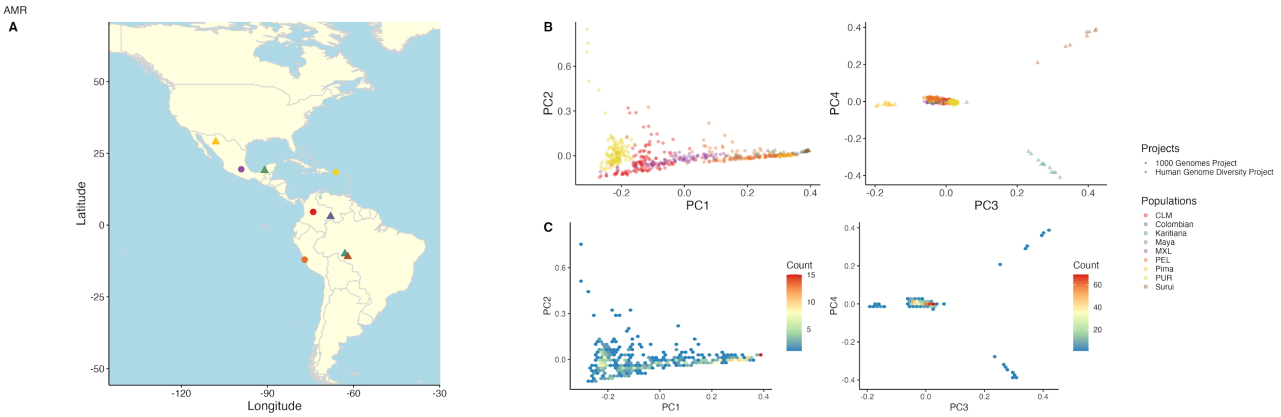

Figure S14 | Subcontinental PCA in AMR populations.
A) Map shows where all AMR samples in analyses are from. B) PCA biplots of PCs 1-4. PCA outliers were removed prior to this analysis. Filled circles indicate populations in the 1000 Genomes Project, while filled triangles indicate populations in HGDP. Population codes are as in **Table S1**. C) Density plot of PCA biplots of PCs 1-4.

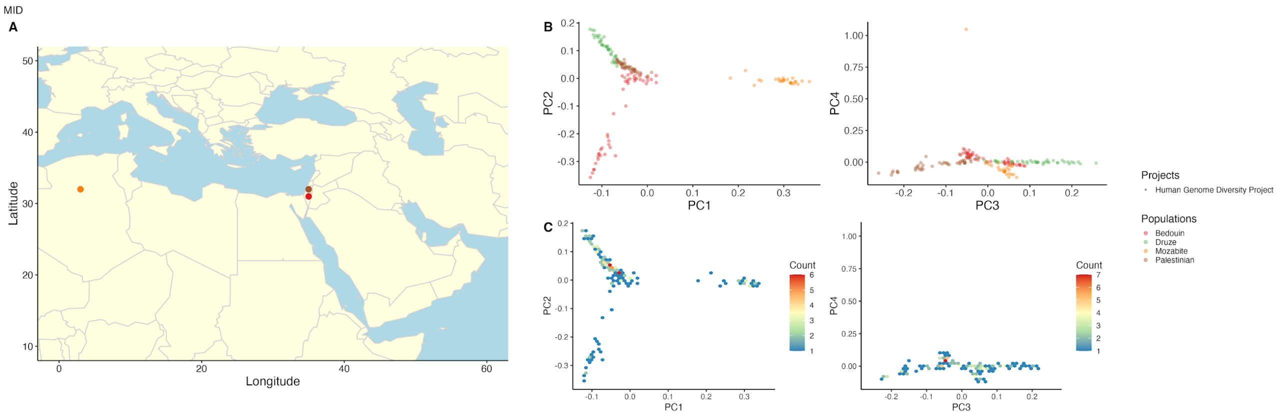

Figure S15 | Subcontinental PCA in MID populations.
A) Map shows where all MID samples in analyses are from. B) PCA biplots of PCs 1-4. PCA outliers were removed prior to this analysis. Filled circles indicate populations in the 1000 Genomes Project, while filled triangles indicate populations in HGDP. Population codes are as in **Table S1**. C) Density plot of PCA biplots of PCs 1-4.

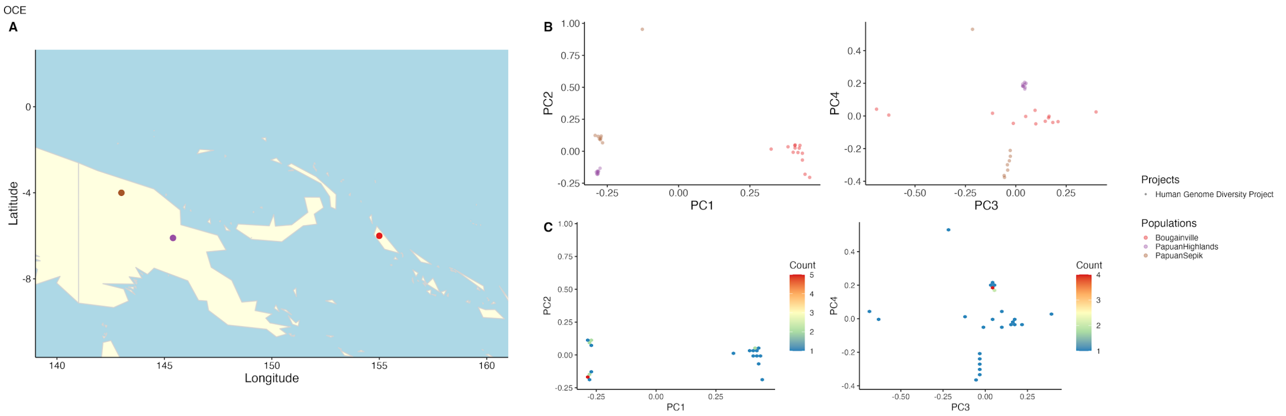

Figure S16 | Subcontinental PCA in OCE populations.
A) Map shows where all OCE samples in analyses are from. B) PCA biplots of PCs 1-4. PCA outliers were removed prior to this analysis. Filled circles indicate populations in the 1000 Genomes Project, while filled triangles indicate populations in HGDP. Population codes are as in **Table S1**. C) Density plot of PCA biplots of PCs 1-4.

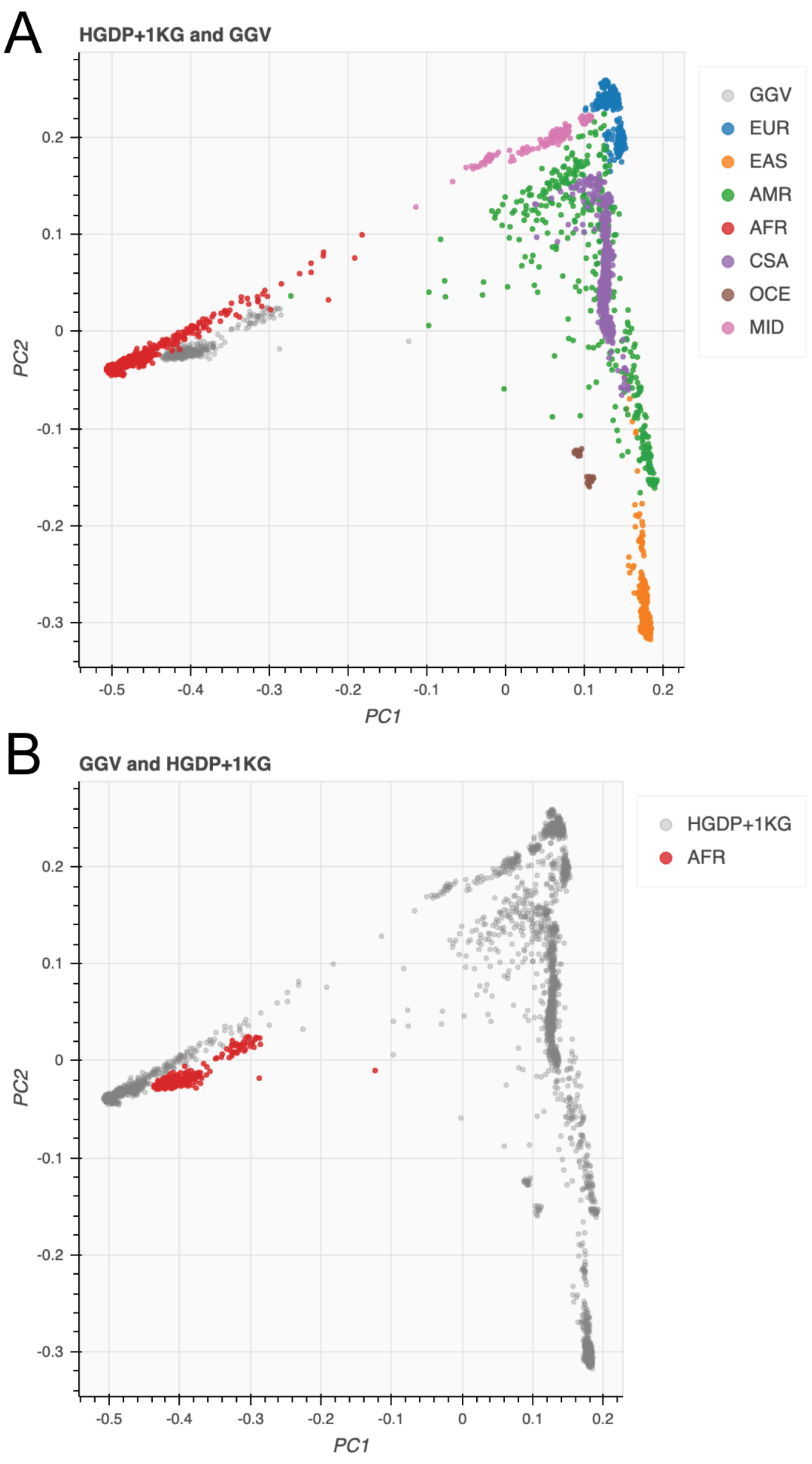

Figure S17 | HGDP+1kGP ancestry labels applied to the Gambian Genome Variation (GGV) Project. A) PCs 1 and 2 of all HGDP+1kGP samples with GGV projected into the same PC space, with each reference population colored and the GGV samples shown in grey. B) The same PCs with the reference data shown in grey and the GGV samples showing the assigned ancestry–all AFR.

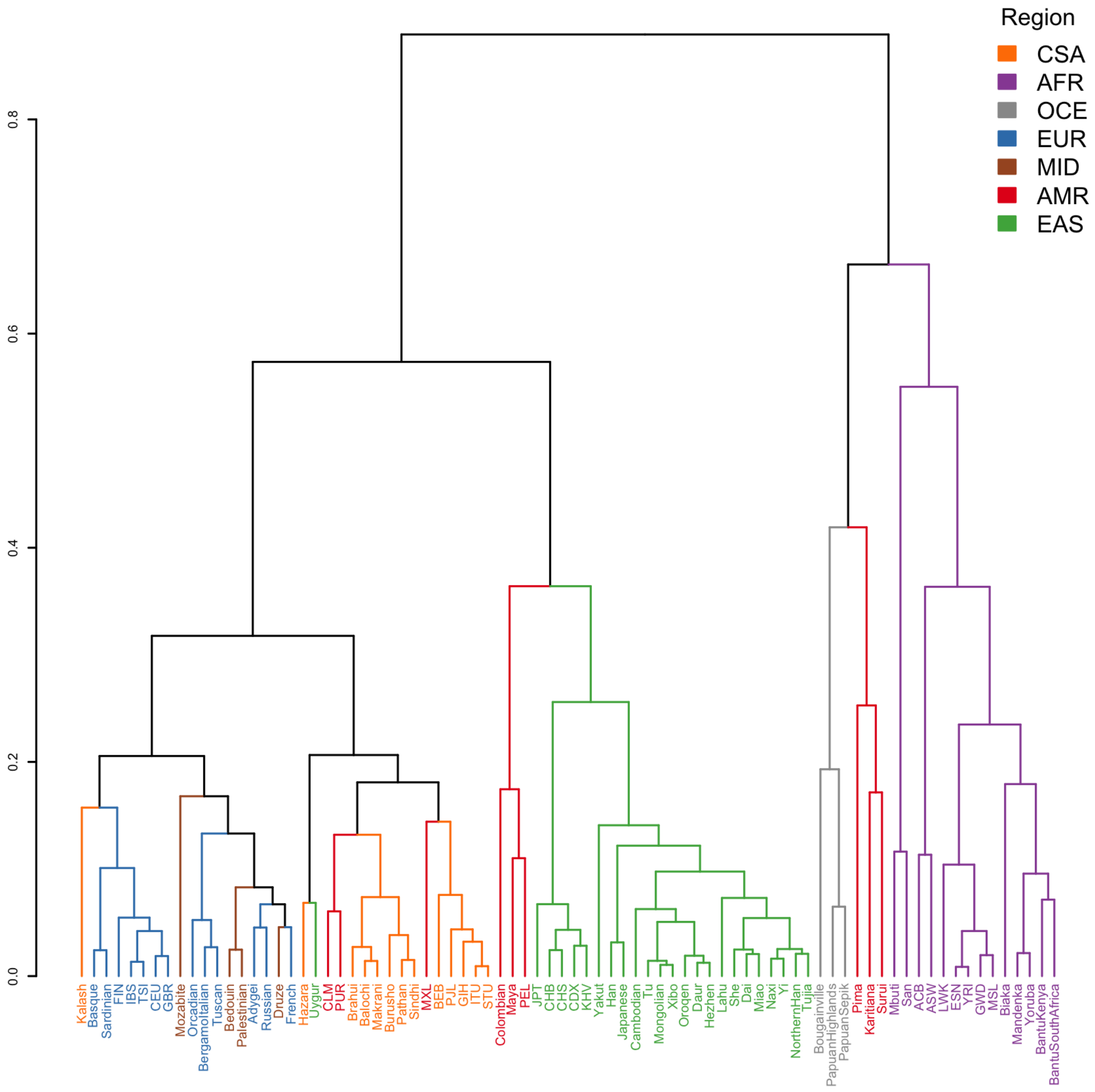

Figure S18 | Dendrogram of the pairwise F_{ST} heatmap between populations colored by geographical/genetic regions.
Populations largely cluster by region with a few exceptions. AMR and MID populations for example are interspersed among other regions.

Table S7 | Populations interspersed among other geographical/genetic regions obtained from the pairwise F_{ST} heatmap, colored by region.

Population	Region	Cluster
Kalash	CSA	CSA population among EUR cluster
Basque	EUR	EUR population cluster
Sardinian	EUR	
FIN	EUR	
IBS	EUR	
TSI	EUR	
CEU	EUR	
GBR	EUR	
Mozabite	MID	MID population among EUR cluster
Orcadian	EUR	EUR population cluster
BergamoItalian	EUR	
Tuscan	EUR	
Bedouin	MID	2 MID populations among EUR cluster
Palestinian	MID	
Adygei	EUR	EUR population cluster
Russian	EUR	
Druze	MID	MID population among EUR cluster
French	EUR	Part of EUR population cluster
Hazara	CSA	Part of CSA population cluster
Uygur	EAS	EAS population among CSA cluster
CLM	AMR	2 AMR populations among CSA cluster
PUR	AMR	
Brahui	CSA	CSA population cluster
Balochi	CSA	
Makrani	CSA	
Burusho	CSA	
Pathan	CSA	
Sindhi	CSA	
MXL	AMR	AMR population among CSA cluster
BEB	CSA	CSA population cluster
PJL	CSA	
GIH	CSA	
ITU	CSA	
STU	CSA	
Colombian	AMR	AMR population cluster
Maya	AMR	
PEL	AMR	
JPT	EAS	EAS population cluster
CHB	EAS	
CHS	EAS	
CDX	EAS	
KHV	EAS	
Yakut	EAS	
Han	EAS	

Japanese	EAS	
Cambodian	EAS	
Tu	EAS	
Mongolian	EAS	
Xibo	EAS	
Oroqen	EAS	
Daur	EAS	
Hezhen	EAS	
Lahu	EAS	
She	EAS	
Dai	EAS	
Miao	EAS	
Naxi	EAS	
Yi	EAS	
NorthernHan	EAS	
Tujia	EAS	
Bougainville	OCE	OCE population cluster
PapuanHighlands	OCE	
PapuanSepik	OCE	
Pima	AMR	AMR population cluster
Karitiana	AMR	
Surui	AMR	
Mbuti	AFR	AFR population cluster
San	AFR	
ACB	AFR	
ASW	AFR	
LWK	AFR	
ESN	AFR	
YRI	AFR	
GWD	AFR	
MSL	AFR	
Biaka	AFR	
Mandenka	AFR	
Yoruba	AFR	
BantuKenya	AFR	
BantuSouthAfrica	AFR	

Table S8 | Pearson's correlation and Mantel tests results with and without waypoints.
Waypoints and calculations are described further in Methods ("F_{ST} versus geographical distance").

Projects	With waypoints		Without waypoints	
	Pearson's correlation coefficient	Mantel statistic	Pearson's correlation coefficient	Mantel statistic
HGDP	0.7615882	0.5471567	0.7095223	0.4504546
1kGP	0.3672138	0.1168814	0.327703	0.1009129
Cross-project	0.450008	0.1925107	0.4119236	0.1549286
Everything	0.5606575	0.3084334	0.5290618	0.2592783

Quality control

Our sample QC procedure was mostly the same as in gnomAD, but differed slightly. Specifically, because whole populations were removed from gnomad 'fail_' filters, we did not filter on the basis of these, which were used in gnomAD v3.1. The clearest example of filters that failed was the fail_n_snp_residual filter, as shown in **Figure S19**.

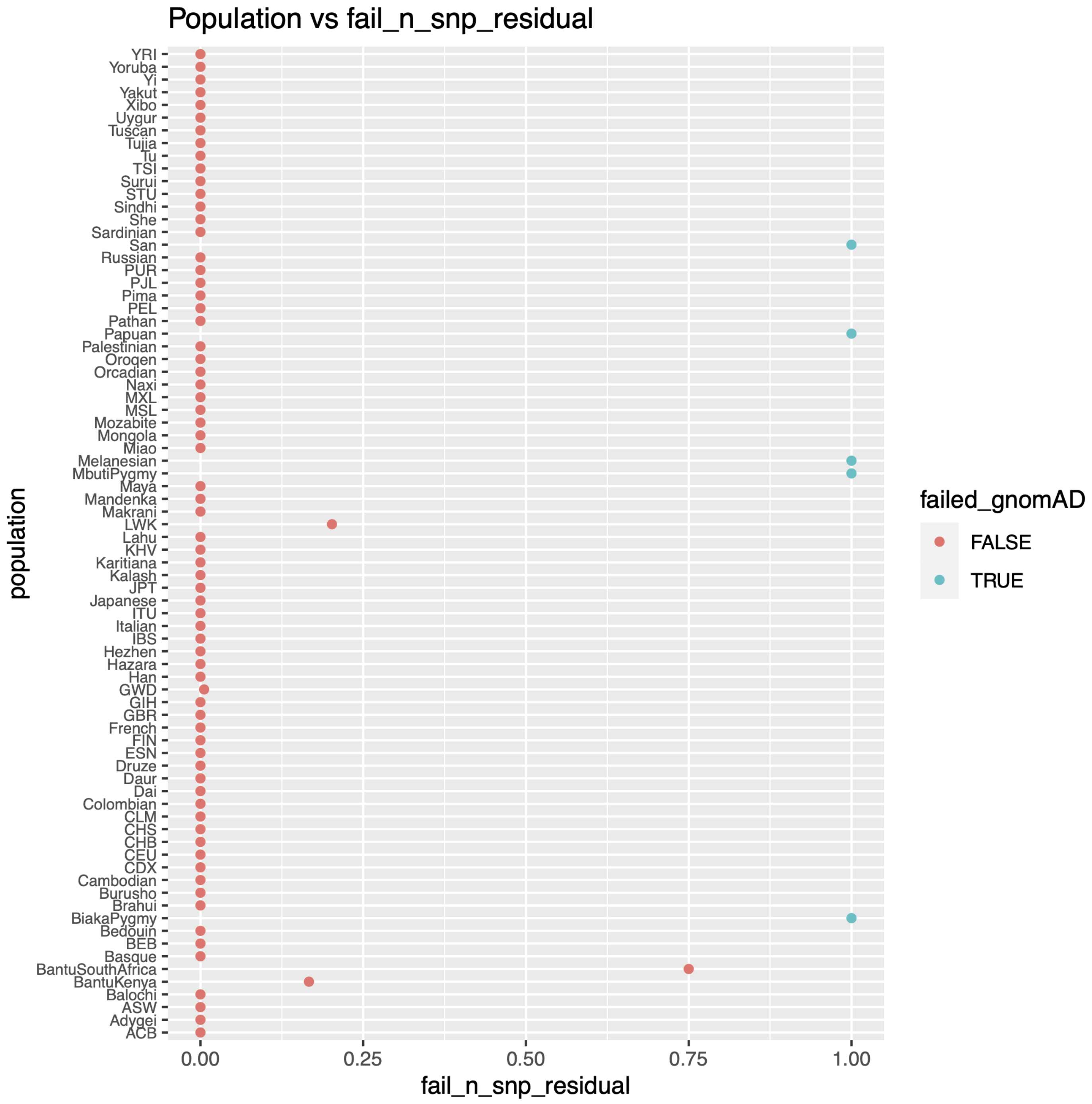

Figure S19 | Example of a filter that was included in gnomAD v3.1 but excluded from this project. The “fail_n_snp_residual” filter, which regresses out principal components from the number of SNPs in an effort to identify technical outliers, would have excluded whole continental groups and populations in this resource because these groups are distinct from the majority of individuals in gnomAD.

Analysis tutorials

To show examples of how to use the individual-level data in a cloud-computing environment, we have created a series of tutorials in iPython notebooks that make use of Hail. These tutorials show how to merge datasets, apply sample and variant QC, run ancestry analysis via PCA and visualization, generate summary statistics of genomes by population, compute and plot population divergence statistics via F_{ST} and F_2 statistics, and intersect external datasets with this dataset and infer ancestry information using project meta-data. The organization of these notebooks is outlined in **Figure 5**.

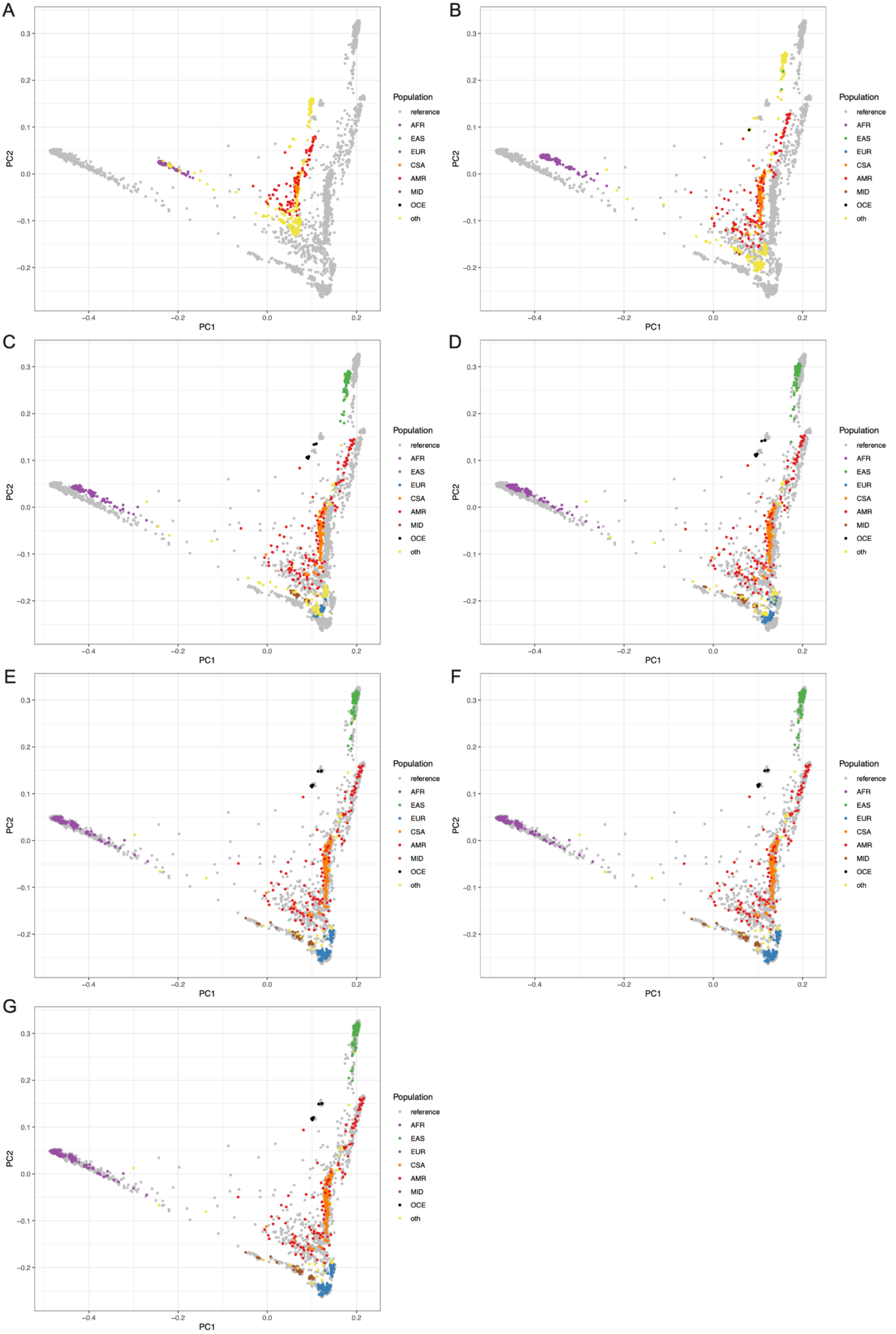
A
B
C
D
E
F
G
PC1
PC2
−0.4
−0.2
0.0
0.2
0.3
0.2
0.1
0.0
−0.1
−0.2
Population
reference
AFR
EAS
EUR
CSA
AMR
MID
OCE
oth

Figure S20 | PCA shrinkage analysis to determine acceptable levels of missingness before ancestry resolution becomes too low to accurately assign population labels.
We started with a set of SNPs that were used in other PCA (e.g. Figure 2), which had undergone LD pruning, minor allele frequency filtering, and missingness filtering. We randomly selected 80% of samples (N=2,704) to train the random forest with corresponding meta-data labels as usual and held out 20% of samples as a test dataset (N=676). After filtering out monomorphic sites from the training dataset once samples were divided, we retained 248,634 variants which were used to train the random forest. We randomly downsampled SNPs in the test dataset to include A) 50%, B) 80%, C) 90%, D) 95%, E) 99%, F) 99.9%, and G) 100% of SNPs in the training dataset. A-G) shows the corresponding projected PCs in the test dataset, showing the extent to which shrinkage affects analyses. **Table S9** shows rates of unclassified individuals by SNP missingness in the test dataset.

Table S9 | Shrinkage analysis matches and no classification numbers by SNP missingness in the test dataset, as shown in Figure S20.
There were no mismatched labels assigned.

Fraction of SNPs in test dataset out of training dataset	Match	No assignment
1	651 / 676 = 0.96	25 / 676 = 0.04
0.999	652 / 676 = 0.96	24 / 676 = 0.04
0.99	649 / 676 = 0.96	27 / 676 = 0.04
0.95	616 / 676 = 0.91	60 / 676 = 0.09
0.9	556 / 676 = 0.82	120 / 676 = 0.18
0.8	447 / 676 = 0.66	229 / 676 = 0.34
0.5	122 / 676 = 0.18	554 / 676 = 0.82

References

1. Chen, S. *et al.* A genome-wide mutational constraint map quantified from variation in 76,156 human genomes. *bioRxiv* 2022.03.20.485034 (2022) doi:10.1101/2022.03.20.485034.
2. Hail Team. *Hail*. (2021). doi:10.5281/zenodo.4504325.
3. 1000 Genomes Project Consortium *et al.* A global reference for human genetic variation. *Nature* **526**, 68–74 (2015).
4. Bergström, A. *et al.* Insights into human genetic variation and population history from 929 diverse genomes. *Science* **367**, (2020).
5. Li, H. *et al.* A synthetic-diploid benchmark for accurate variant-calling evaluation. *Nat. Methods* **15**, 595–597 (2018).
