# Supplementary material for: A harmonized public resource of deeply sequenced diverse human genomes
Source: bioRxiv. 2023 Feb 2:2023.01.23.525248. Preprint. [Version 2] doi: 10.1101/2023.01.23.525248 (PMC9900804; doi:10.1101/2023.01.23.525248)
Supplement: Supplementary file 1 [file media-1.pdf]

HGDP+1kG supplement

HGDP+1kG supplement **1**
Data harmonization 3
Genotype data 3
Metadata 3
Table S1 | Harmonization of HGDP and 1000 Genomes Project meta-data project labels. These labels are referred to as geographical/genetic region throughout this manuscript. 3
Table S2 | Genetic outliers identified in analysis of global and subcontinental PCA. 3
Table S3 | Final sample counts. Note: hard filtering was performed as in gnomAD v3 with modifications as described b (in the initial gnomAD release, 3,280 of these 4,120 hard filtered individuals are included). CHM = Complete Hydatidiform Mole described previously 5. 4
QC Metadata Summaries 4
Figure S1 | Coverage across the 1kGP and HGDP. Coverage in both datasets is uniformly above 30X, with an average of 33X coverage across the harmonized dataset. The coverage of the HGDP genomes is more variable than in 1kGP, as expected based on a variety of technical differences such as multiple sequencing batches, PCR+ vs PCR-free, and older cell lines in HGDP compared to 1kGP. The differences in project coverages also impacts the distribution of coverage statistics by Geographical region given their tally by project (Table S4). The overall coverage distributions by population are shown in Figure S2. 5
Figure S2 | Coverage across 1kGP and HGDP by population. Regional abbreviations are as described in Table S1. OCE is excluded from this plot as it is represented by only two populations. Mean coverage across the different regions is 33X with coverage consistently above 30X for all regions. 5
Table S4 | Coverage and SNV statistics by population. 5
Structural variants (SVs) 6
Figure S3. Dosage and sex ploidy of HGDP samples and batching strategy. A) Distribution of dosage scores across HGDP samples. We used the previously developed whole genome dosage model (Collins et al 2020) to quantify non-uniform distribution of sequencing coverage.The dosage scores corresponded predominantly to PCR-amplified (PCR+) and PCR-free (PCR-) library protocols. B) Distribution of chrX copy number across HGDP samples. C) Batching strategy for SV calling. HGDP samples were first split by their PCR status and chrX ploidy. PCR- samples were then ranked by their sequencing depth from low to high, and split into four sub batches of equivalent sizes. Male and female batches with matched coverage quantiles are combined to form the final batches. 6
Figure S4 | SV callset and quality evaluation results. A) Count of SV sites across 4,150 HGDP and 1KGP samples by variant type. B) Count of SVs per genome by variant type. C) Count of SV sites by allele frequency. D) Inheritance of SVs calculated in 100 pather-mother-child trio families. E) Correlation of allele frequencies. F) Hardy-Weinberg Equilibrium distribution of SVs. 7
Table S5 | Sex chromosome aneuploidies in the HGDP samples. 7
Figure S5 | Mean count of SVs versus SNVs by project, region, and number of individuals. Top line shows a fitted regression line to the 1000 Genomes Project points, and bottom line is fitted to HGDP points. A larger number of SVs are present in the 1000 Genomes Project data, which was explored more fully in Figure S6. 8
Table S6 | SV calls by external support from HGSV study. 8
Figure S6 | SV breakdown in count by class across HGDP and 1kGP (HGSV). Per genome SV counts by study and PCR status (A,C), and population (B). Per genome SV counts are also broken

down by SV type, including deletions, duplications, multi-allelic CNVs, insertions, inversions, and complex SVs in D). 9
Population genetic comparisons 10
Figure S7 | ADMIXTURE analysis of the HGDP and 1kGP resource. We ran ADMIXTURE with values of K=2 through K=10 across populations and harmonized geographical/genetic regions. Each row of bar plots shows the breakdown of regional substructure as K increases, where K is the number of genetic ancestry components fit in that run. For example, when K=2, AFR separates from the rest of the populations as the most distinct population due to high levels of genetic diversity. When K=3 EUR separates from the rest, and so on. We chose the best fit value of K to be K=6 based on a reduction in the rate of change of 5-fold cross validation error as shown in Figure S8. 12
Figure S8 | 5-fold cross-validation error across ADMIXTURE runs. We selected K=6 as the point at which cross-validation error leveled out. As described in the ADMIXTURE manual, the cross-validation error enables users to identify the value of K for which the model has best predictive accuracy, as determined by “holding out” data points. It partitions observed genotypes into 5 roughly equally sized folds, masks genotypes for each fold, then predicts the genotypes. 12
Figure S9 | Subcontinental PCA of each geographical/genetic region. Each row shows PCA biplots of PCs 1-4. PCA outliers were removed prior to this analysis. Filled circles indicate populations in the 1000 Genomes Project, while filled triangles indicate populations in HGDP. Population codes are as in Table S1. 14
Figure S10 | HGDP+1kGP ancestry labels applied to the Gambian Genome Variation (GGV) Project. A) PCs 1 and 2 of all HGDP+1kGP samples with GGV projected into the same PC space, with each reference population colored and the GGV samples shown in grey. B) The same PCs with the reference data shown in grey and the GGV samples showing the assigned ancestry–all AFR. 15
Quality control 15
Figure S11 | Example of a filter that was included in gnomAD v3.1 but excluded from this project. The “fail_n_snp_residual” filter, which regresses out principal components from the number of SNPs in an effort to identify technical outliers, would have excluded whole continental groups and populations in this resource because these groups are distinct from the majority of individuals in gnomAD. 16
Analysis tutorials 16
Figure S13 | PCA shrinkage analysis to determine acceptable levels of missingness before ancestry resolution becomes too low to accurately assign population labels. We started with a set of SNPs that were used in other PCA (e.g. Figure 2), which had undergone LD pruning, minor allele frequency filtering, and missingness filtering. We randomly selected 80% of samples (N=2,704) to train the random forest with corresponding meta-data labels as usual and held out 20% of samples as a test dataset (N=676). After filtering out monomorphic sites from the training dataset once samples were divided, we retained 248,634 variants which were used to train the random forest. We randomly downsampled SNPs in the test dataset to include A) 50%, B) 80%, C) 90%, D) 95%, E) 99%, F) 99.9%, and G) 100% of SNPs in the training dataset. A-G) shows the corresponding projected PCs in the test dataset, showing the extent to which shrinkage affects analyses. Table S7 shows rates of unclassified individuals by SNP missingness in the test dataset. 19
Table S7 | Shrinkage analysis matches and no classification numbers by SNP missingness in the test dataset, as shown in Figure S13. There were no mismatched labels assigned. 19
References 19

Data harmonization

Genotype data

Genotype data was processed as described in [1]. Briefly, reads were mapped using BWA-MEM, cleaned using the GATK Best Practices pipeline, and gVCFs were generated using GATK HaplotypeCaller. Joint calling was performed using the Hail combiner [2] and converted to a VariantDataset (VDS), which was then densified into a dense MatrixTable used for analysis. These datasets are released on Google Cloud Platform, Amazon Web Services, and Microsoft Azure, and can be found on the Downloads page of the gnomAD browser (https://gnomad.broadinstitute.org/downloads#v3-hgdp-1kg).

Metadata

Where possible, we combined meta-data from the 1000 Genomes Project and HGDP by combining the "super population" data from the 1000 Genomes project [3] and region information from HGDP [4]. We created a harmonized combined label with 3-letter codes for all groups, which we refer to as geographical/genetic region throughout the text. Where a region was only clearly contained in HGDP, we used the HGDP information to define a 3-letter code. The CENTRAL_SOUTH_ASIA code contained within HGDP is more geographically expansive than the SAS label contained in the 1000 Genomes Project, so we expanded the 3 letter code to be CSA, as shown in **Table S1**.

Table S1 | Harmonization of HGDP and 1000 Genomes Project meta-data project labels. These labels are referred to as geographical/genetic region throughout this manuscript.

1000 Genomes super population	HGDP region	Combined label (geographical/genetic region)
AFR	AFRICA	AFR
AMR	AMERICA	AMR
SAS	CENTRAL_SOUTH_ASIA	CSA
EAS	EAST_ASIA	EAS
EUR	EUROPE	EUR
N/A	MIDDLE_EAST	MID
N/A	OCEANIA	OCE

After combining region data, we then used principal components analysis (PCA) to identify ancestry outliers within regions. We identified outliers as described in **Table S2** and provide final sample counts in **Table S3**.

Table S2 | Genetic outliers identified in analysis of global and subcontinental PCA.

Sample ID	Region	Population
HG01880	AFR	ACB
HG01881	AFR	ACB
NA20274	AFR	ASW
NA20299	AFR	ASW

NA20314	AFR	ASW
HGDP00013	CSA	Brahui
HGDP00029	CSA	Brahui
HGDP00057	CSA	Balochi
HGDP00130	CSA	Makrani
HGDP00150	CSA	Makrani
HGDP00175	CSA	Sindhi
HGDP01298	EAS	Uygur
HGDP01300	EAS	Uygur
HGDP01303	EAS	Uygur
LP6005443-DNA_B02	EAS	Uygur
HG01628	EUR	IBS
HG01629	EUR	IBS
HG01630	EUR	IBS
HG01694	EUR	IBS
HG01696	EUR	IBS
HGDP00621	MID	Bedouin
HGDP01270	MID	Mozabite
HGDP01271	MID	Mozabite
CHMI_CHMI3_WGS2	gnomAD QC sample	

Table S3 | Final sample counts. Note: hard filtering was performed as in gnomAD v3 with modifications as described b (in the initial gnomAD release, 3,280 of these 4,120 hard filtered individuals are included). Total in first two rows includes a “synthetic diploid” QC sample (CHM; Complete Hydatidiform Mole) described previously[5].

	HGDP	1kG	Total
Initial dataset	948	3,202	4,151
Hard filtered	943	3,176	4,120
PCA outliers removed	930	3,166	4,096
Unrelated individuals	807	2,507	3,378

QC Metadata Summaries

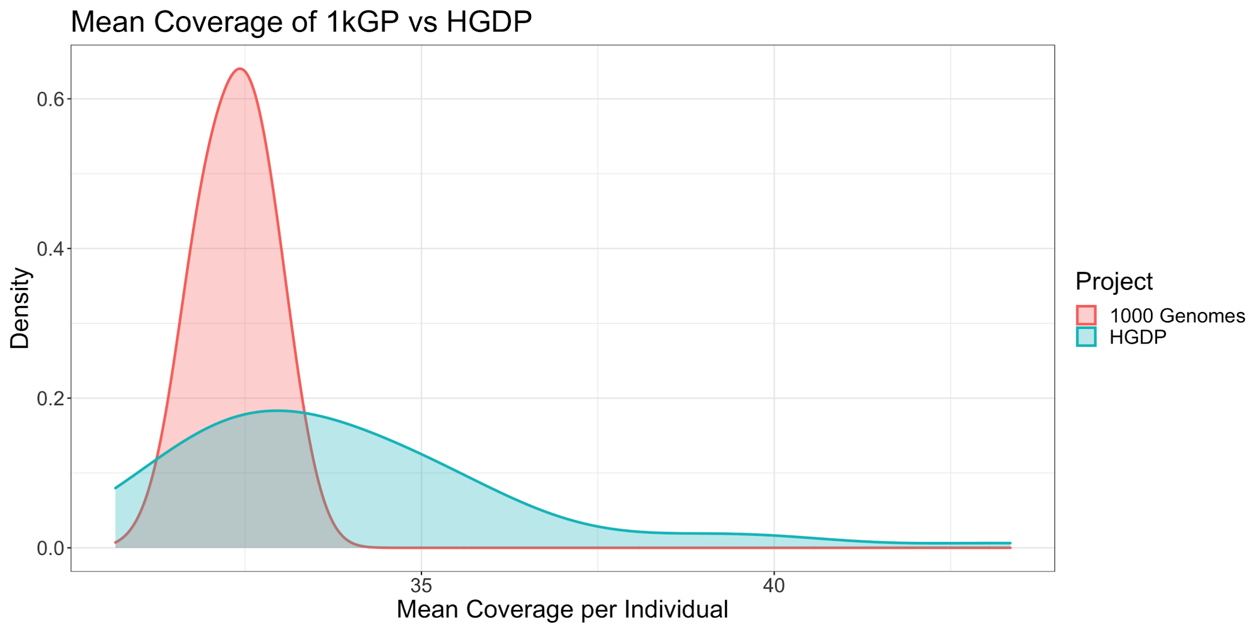

Figure S1 | Coverage across the 1kGP and HGDP. Coverage in both datasets is uniformly above 30X, with an average of 33X coverage across the harmonized dataset. The coverage of the HGDP genomes is more variable than in 1kGP, as expected based on a variety of technical differences such as multiple sequencing batches, PCR+ vs PCR-free, and older cell lines in HGDP compared to 1kGP. The differences in project coverages also impacts the distribution of coverage statistics by Geographical region given their tally by project (**Table S4**). The overall coverage distributions by population are shown in **Figure S2**.

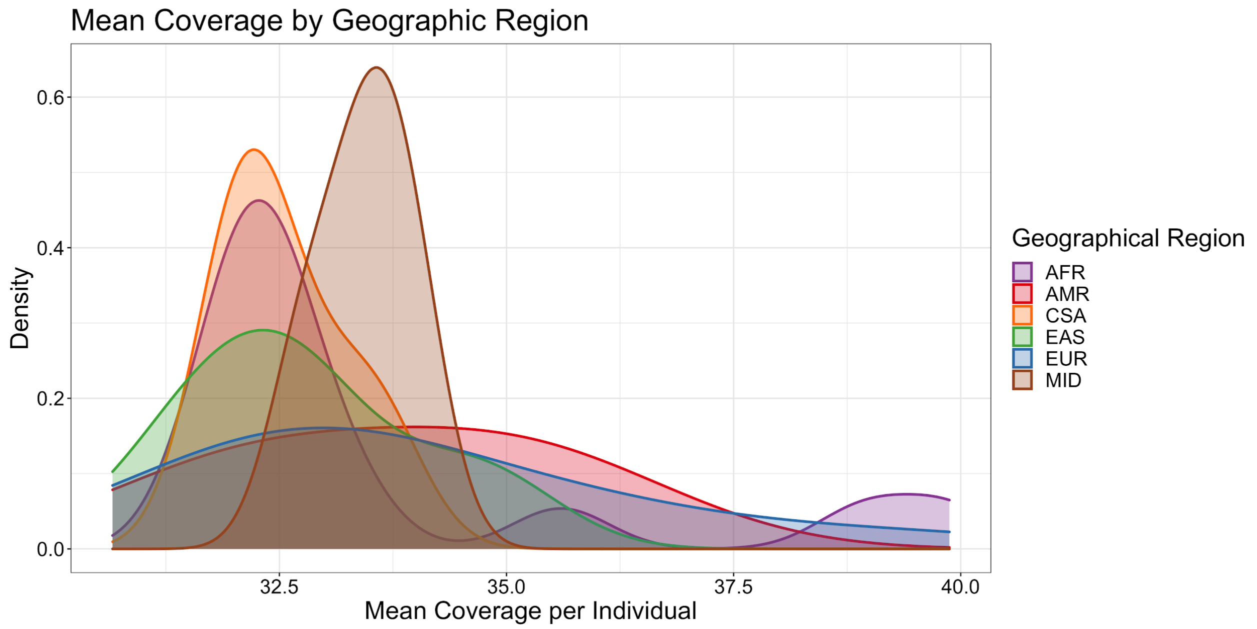

Figure S2 | Coverage across 1kGP and HGDP by population. Regional abbreviations are as described in **Table S1**. OCE is excluded from this plot as it is represented by only two populations. Mean coverage across the different regions is 33X with coverage consistently above 30X for all regions.

Table S4 | Coverage and SNV statistics by population.
Coverage was computed across the genome as part of the gnomAD project. Relatedness was inferred using PC-Relate. Because number of variants and singleton counts per individual are sensitive to sample size imbalances, they were tallied using a downsampled version of the dataset in which each population was

randomly downsampled to match the smallest population (i.e. 6 individuals per population), then SNVs were removed if they were not polymorphic in the downsampled dataset. Given the more pronounced impact of batch effects on structure variant (SV) calling and the number of batches present within and between datasets, the number of SVs per individual were calculated across the full dataset, not in the downsampled dataset.

Structural variants (SVs)

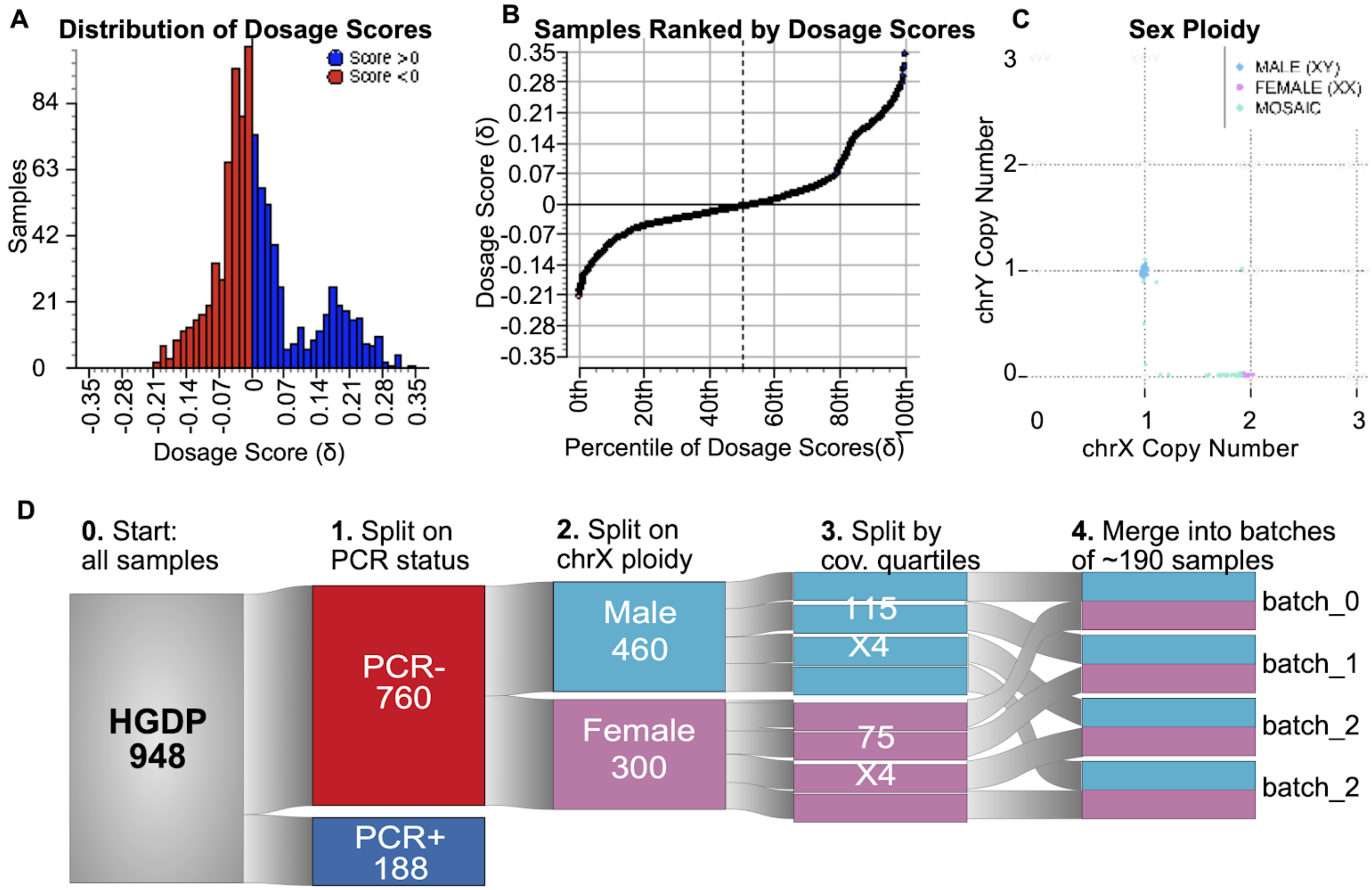

Figure S3. Dosage and sex ploidy of HGDP samples and batching strategy. A) Distribution of dosage scores across HGDP samples. We used the previously developed whole genome dosage model (Collins et al 2020) to quantify non-uniform distribution of sequencing coverage.The dosage scores corresponded predominantly to PCR-amplified (PCR+) and PCR-free (PCR-) library protocols. B) Samples ranked by dosage score. C) Distribution of chrX copy number across HGDP samples. D) Batching strategy for SV calling. HGDP samples were first split by their PCR status and chrX ploidy. PCR- samples were then ranked by their sequencing depth from low to high, and split into four sub batches of equivalent sizes. Male and female batches with matched coverage quantiles are combined to form the final batches.

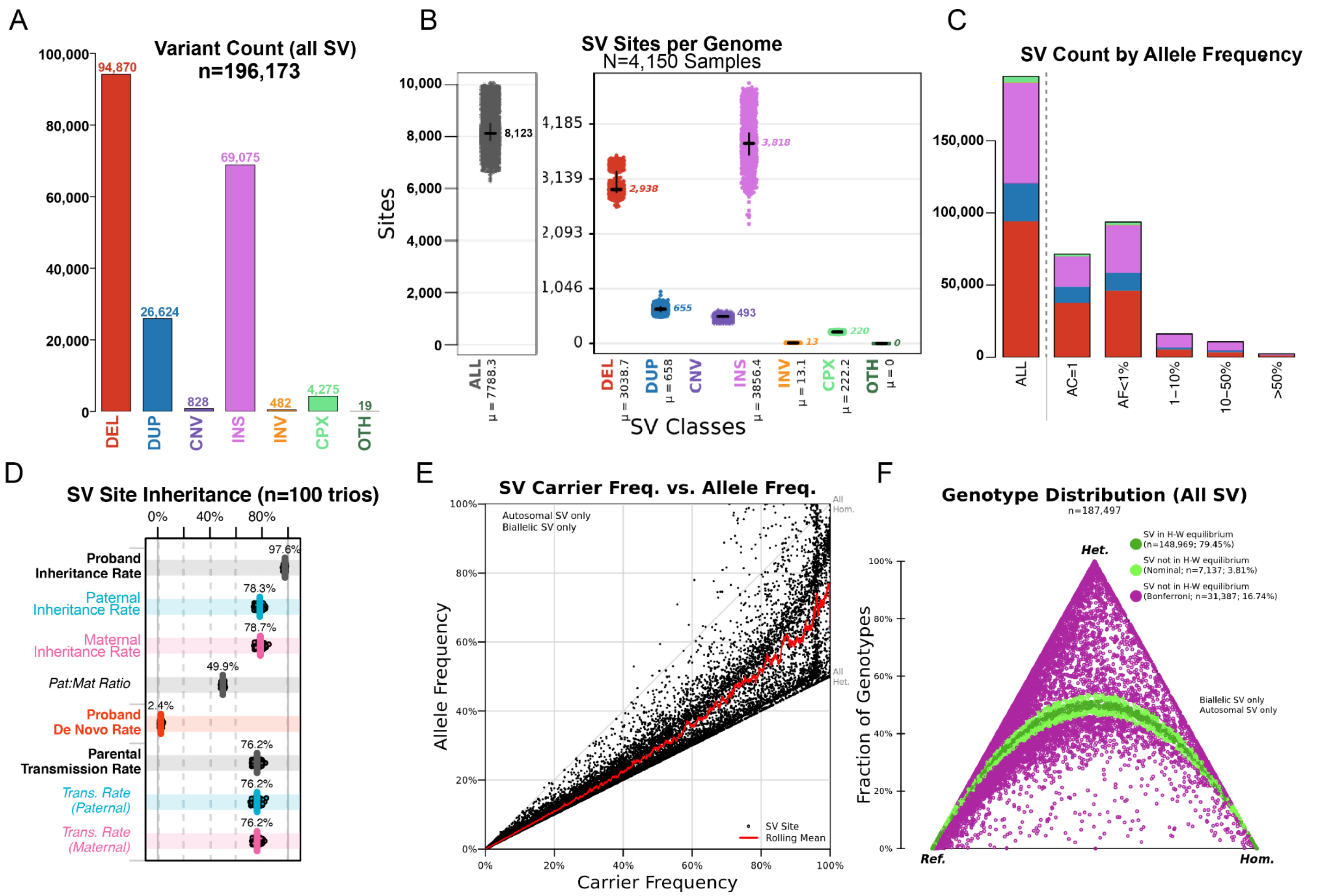

Figure S4 | SV callset and quality evaluation results. A) Count of SV sites across 4,150 HGDP and 1KGP samples by variant type. B) Count of SVs per genome by variant type. C) Count of SV sites by allele frequency. D) Inheritance of SVs calculated in 100 pather-mother-child trio families. E) Correlation of allele frequencies. F) Hardy-Weinberg Equilibrium distribution of SVs.

Table S5 | Sex chromosome aneuploidies in the HGDP samples.

Sample ID	Population	Genetic region	chrX	chrY	Assignment
HGDP00445	Burusho	CSA	1	0	XO
HGDP01157	Bergamo Italian	EUR	1	0	XO
HGDP01208	Oroquen	EAS	2	1	XXY
HGDP01368	Basque	EUR	1	0	XO
LP6005441-DNA_G09	Palestinian	MID	1	0	XO

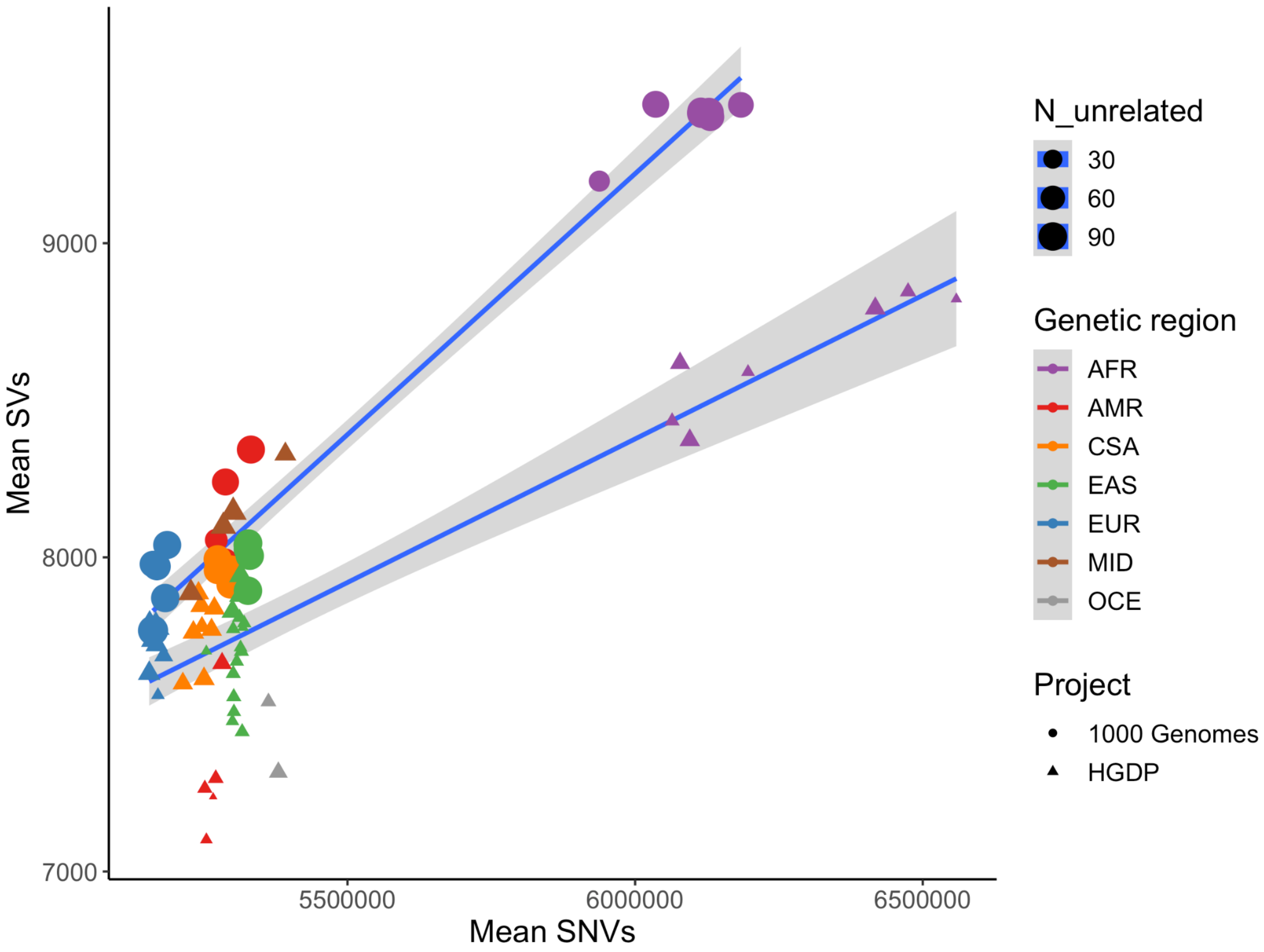

Figure S5 | Mean count of SVs versus SNVs by project, region, and number of individuals. Top line shows a fitted regression line to the 1000 Genomes Project points, and bottom line is fitted to HGDP points. A larger number of SVs are present in the 1000 Genomes Project data, which was explored more fully in **Figure S6**.

Table S6 | SV calls by external support from HGSV study.

		External Supports (Count SVs per genome)			
SVtype	Precision	No Support	Illumina	PacBio	Illumina and PacBio
DEL	97.60%	74	116	205	2688
DUP	89.30%	73	34	216	359
INS	91.37%	346	456	320	2889
INV	85.71%	2	0	12	0
CNV	71.29%	143	12	308	35
CPX	75.89%	54	0	170	0

All-SVs	91.87%	692	618	5971	1231

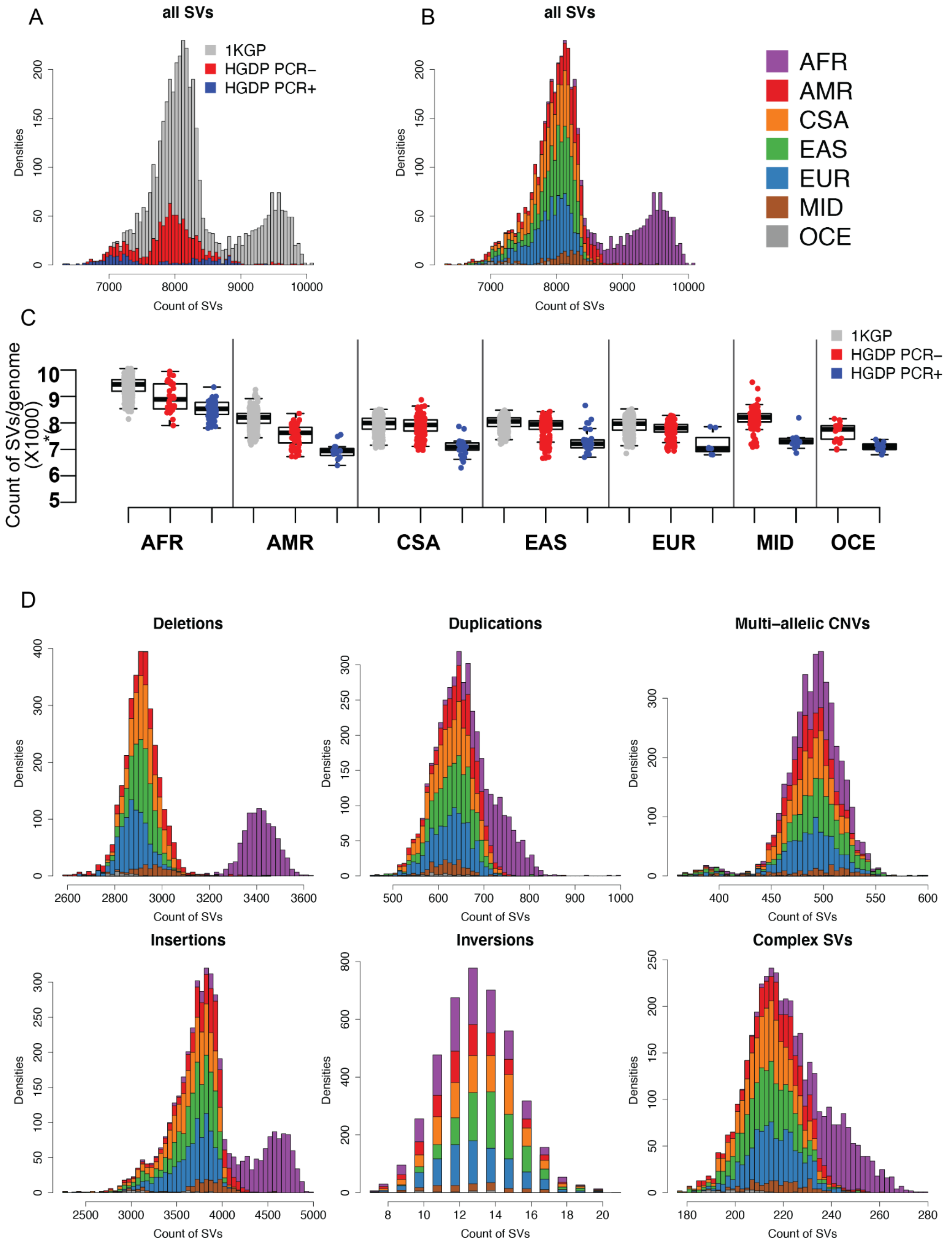

Figure S6 | SV breakdown in count by class across HGDP and 1kGP (HGSV). Per genome SV counts by study and PCR status (A,C), and population (B). Per genome SV counts are also broken down by SV type, including deletions, duplications, multi-allelic CNVs, insertions, inversions, and complex SVs in D).

Population genetic comparisons

The breakdown of ancestry and population structure by ADMIXTURE is similar to that identified in global PCA, with K=2 highlighting structure in the AFR, K=3 highlighting structure in the EAS, K=4 highlighting structure in the EUR and CSA, K=5 highlighting structure in the AMR, K=6 highlighting structure in the OCE, K=7 highlighting structure in the MID, and subsequent values of K highlighting structure within meta-data labels (**Figure S7**).

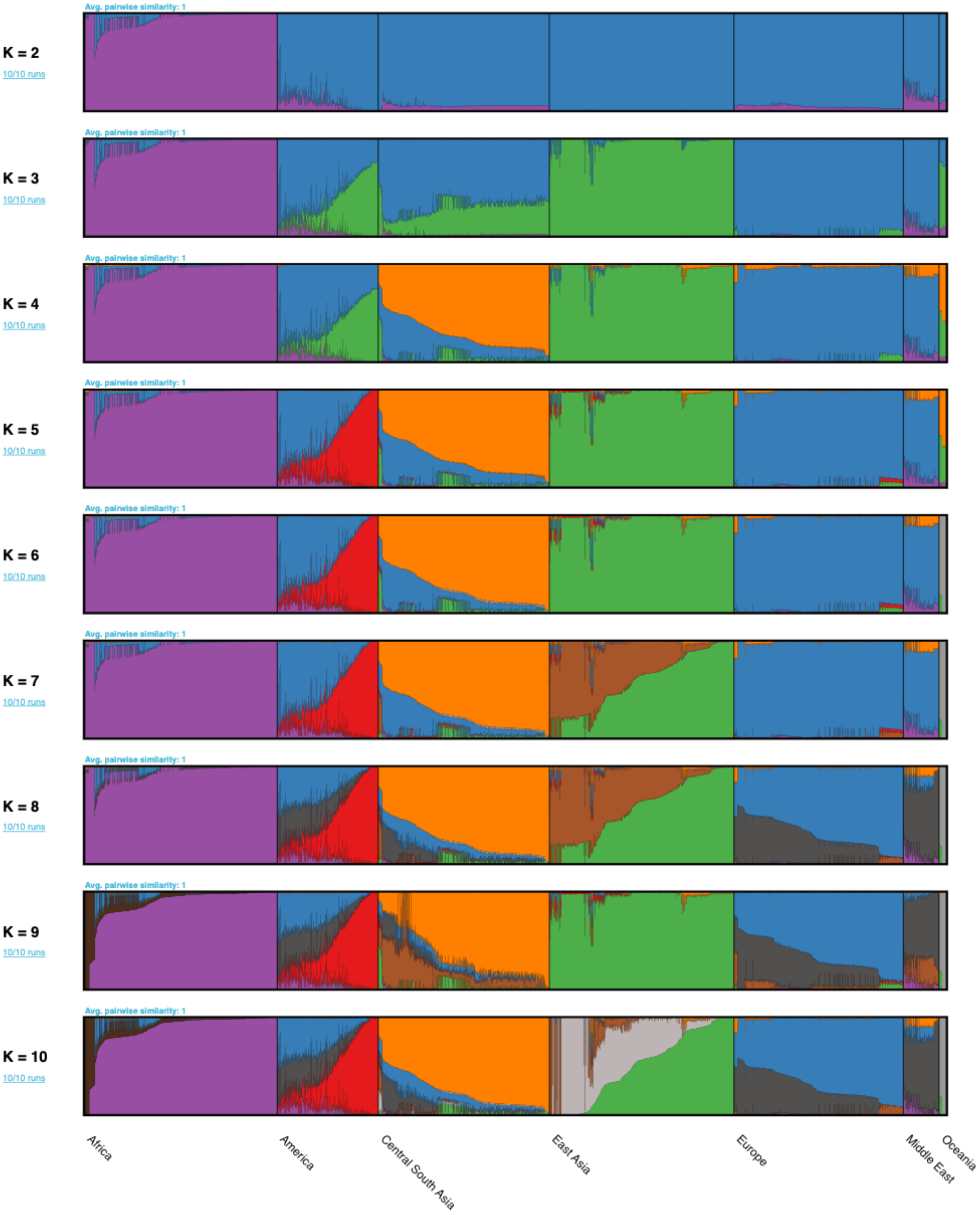
Avg. pairwise similarity: 1
K = 2
10/10 runs
K = 3
K = 4
K = 5
K = 6
K = 7
K = 8
K = 9
K = 10
Africa
America
Central South Asia
East Asia
Europe
Middle East
Oceania

Figure S7 | ADMIXTURE analysis of the HGDP and 1kGP resource. We ran ADMIXTURE with values of K=2 through K=10 across populations and harmonized geographical/genetic regions. Each row of bar plots shows the breakdown of regional substructure as K increases, where K is the number of genetic ancestry components fit in that run. For example, when K=2, AFR separates from the rest of the populations as the most distinct population due to high levels of genetic diversity. When K=3 EUR separates from the rest, and so on. We chose the best fit value of K to be K=6 based on a reduction in the rate of change of 5-fold cross validation error as shown in **Figure S8**.

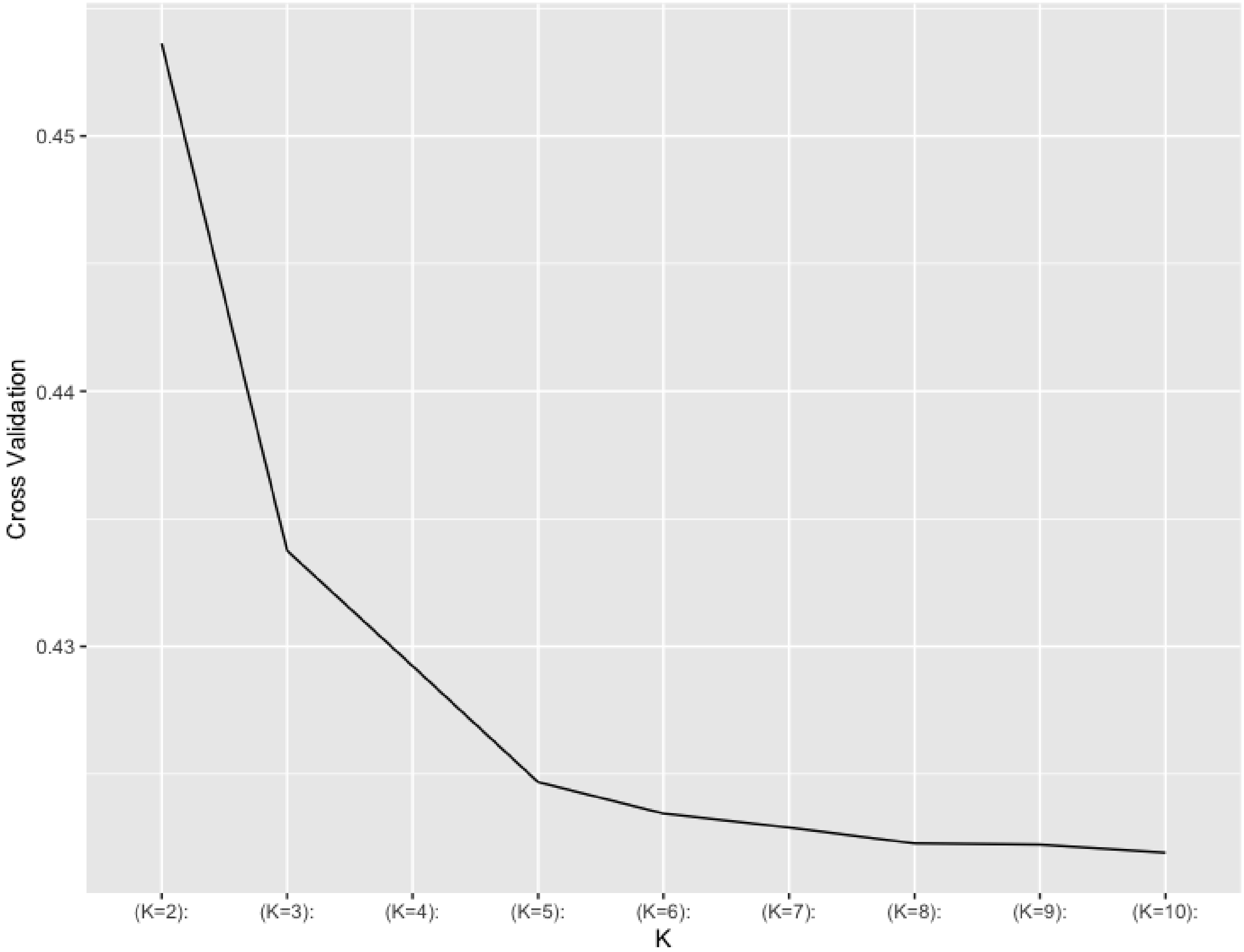

Figure S8 | 5-fold cross-validation error across ADMIXTURE runs. We selected K=6 as the point at which cross-validation error leveled out. As described in the ADMIXTURE manual, the cross-validation error enables users to identify the value of K for which the model has best predictive accuracy, as determined by "holding out" data points. It partitions observed genotypes into 5 roughly equally sized folds, masks genotypes for each fold, then predicts the genotypes.

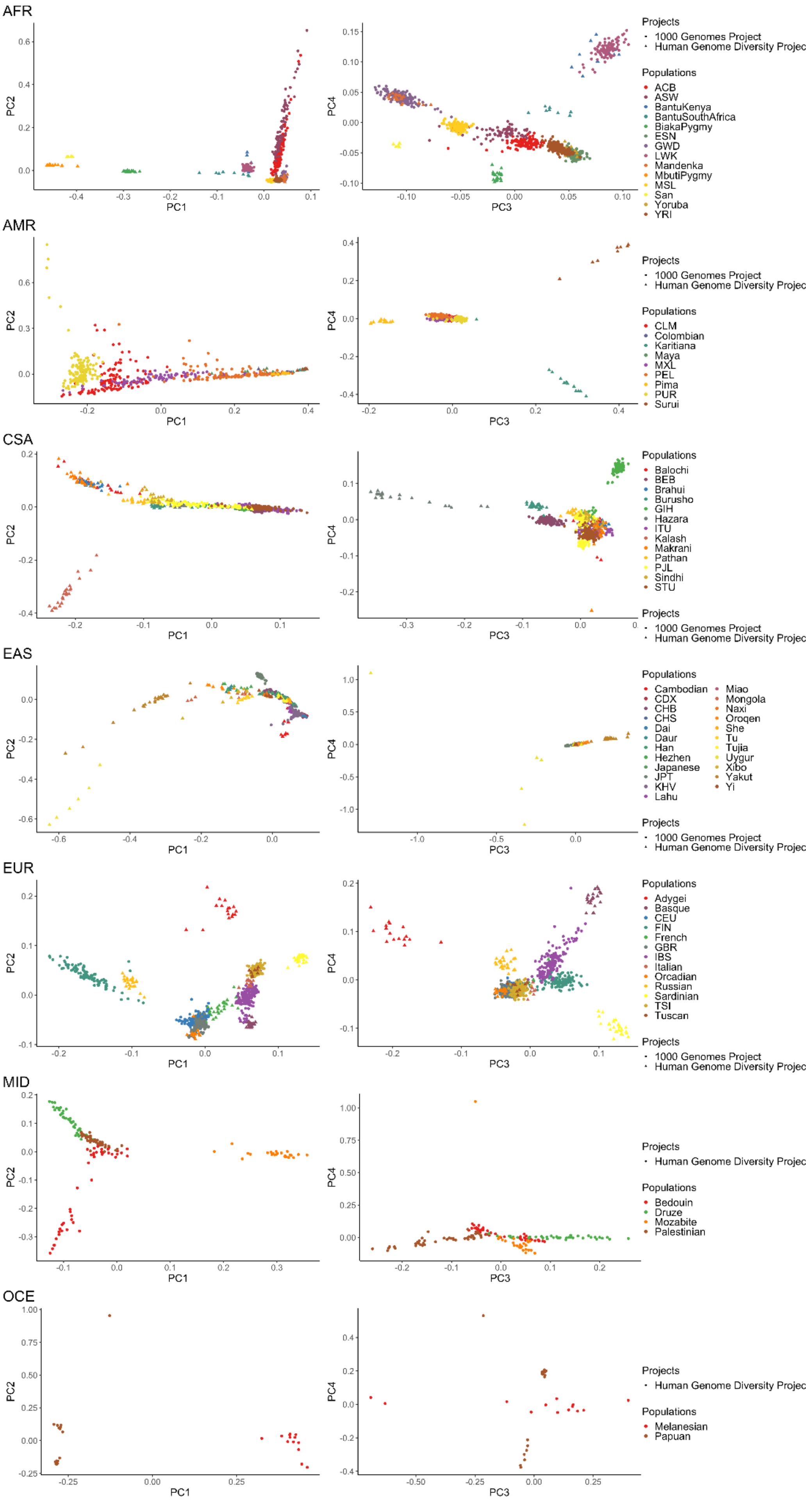
AFR
PC1
PC2
PC3
PC4
Projects
1000 Genomes Project
Human Genome Diversity Projec
Populations
ACB
ASW
BantuKenya
BantuSouthAfrica
BiakaPygmy
ESN
GWD
LWK
Mandenka
MbutiPygmy
MSL
San
Yoruba
YRI
AMR
Projects
1000 Genomes Project
Human Genome Diversity Projec
Populations
CLM
Colombian
Karitiana
Maya
MXL
PEL
Pima
PUR
Surui
CSA
Populations
Balochi
BEB
Brahui
Burusho
GIH
Hazara
ITU
Kalash
Makrani
Pathan
PJL
Sindhi
STU
Projects
1000 Genomes Project
Human Genome Diversity Projec
EAS
Populations
Cambodian
CDX
CHB
CHS
Dai
Daur
Han
Hezhen
Japanese
JPT
KHV
Lahu
Miao
Mongola
Naxi
Oroqen
She
Tu
Tujia
Uygur
Xibo
Yakut
Yi
Projects
1000 Genomes Project
Human Genome Diversity Projec
EUR
Populations
Adygei
Basque
CEU
FIN
French
GBR
IBS
Italian
Orcadian
Russian
Sardinian
TSI
Tuscan
Projects
1000 Genomes Project
Human Genome Diversity Projec
MID
Projects
Human Genome Diversity Projec
Populations
Bedouin
Druze
Mozabite
Palestinian
OCE
Projects
Human Genome Diversity Projec
Populations
Melanesian
Papuan

Figure S9 | Subcontinental PCA of each geographical/genetic region. Each row shows PCA biplots of PCs 1-4. PCA outliers were removed prior to this analysis. Filled circles indicate populations in the 1000 Genomes Project, while filled triangles indicate populations in HGDP. Population codes are as in **Table S1**.

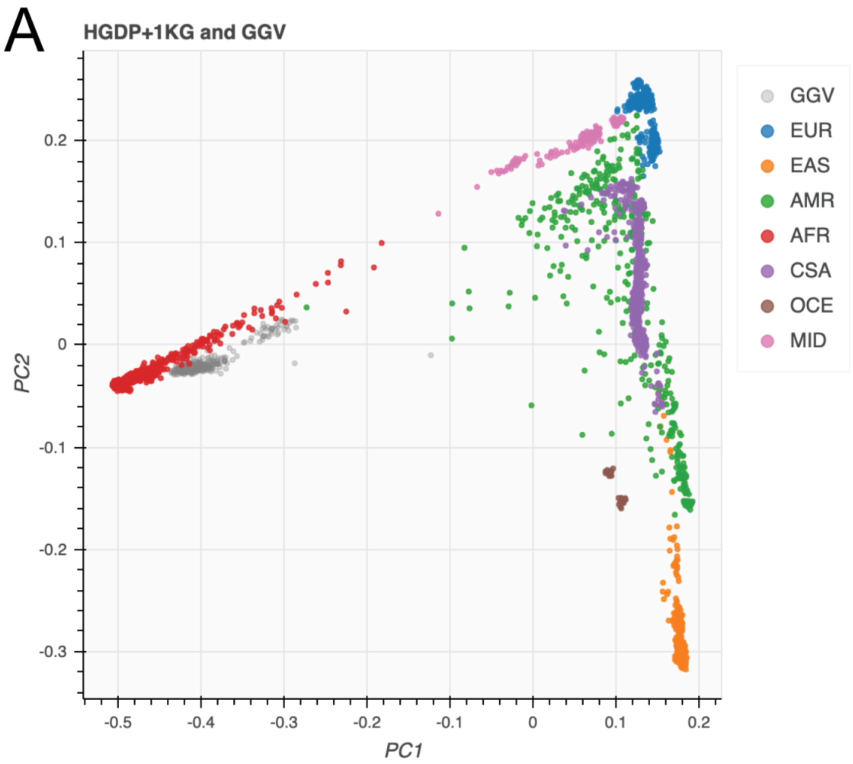

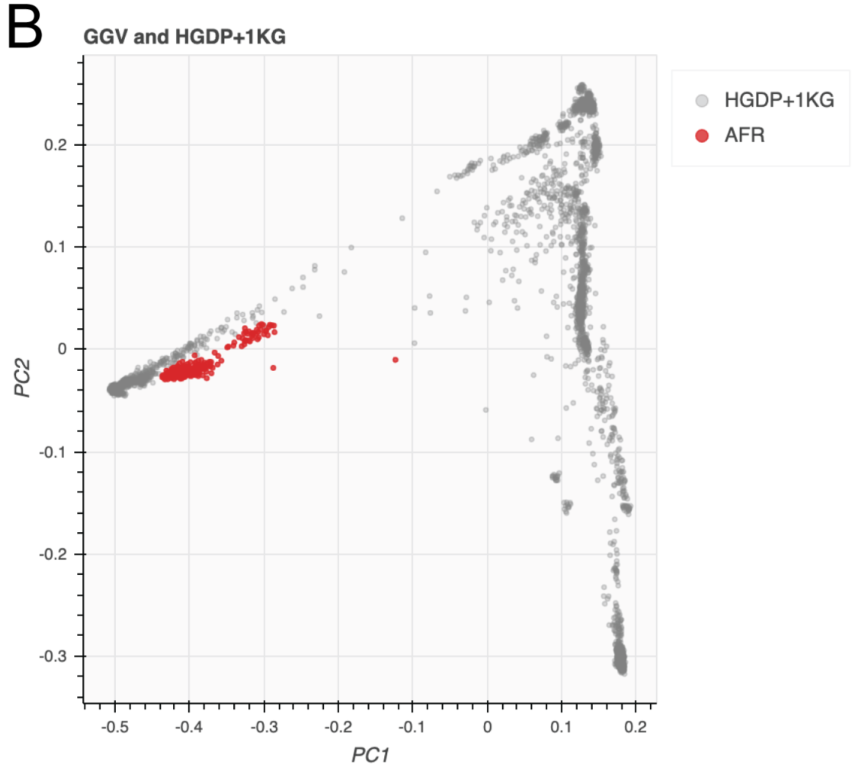

Figure S10 | HGDP+1kGP ancestry labels applied to the Gambian Genome Variation (GGV) Project. A) PCs 1 and 2 of all HGDP+1kGP samples with GGV projected into the same PC space, with each reference population colored and the GGV samples shown in grey. B) The same PCs with the reference data shown in grey and the GGV samples showing the assigned ancestry–all AFR.

Quality control

Our sample QC procedure was mostly the same as in gnomAD, but differed slightly. Specifically, because whole populations were removed from gnomad 'fail_' filters, we did not filter on the basis of these, which were used in gnomAD v3.1. The clearest example of filters that failed was the fail_n_snp_residual filter, as shown in **Figure S11**.

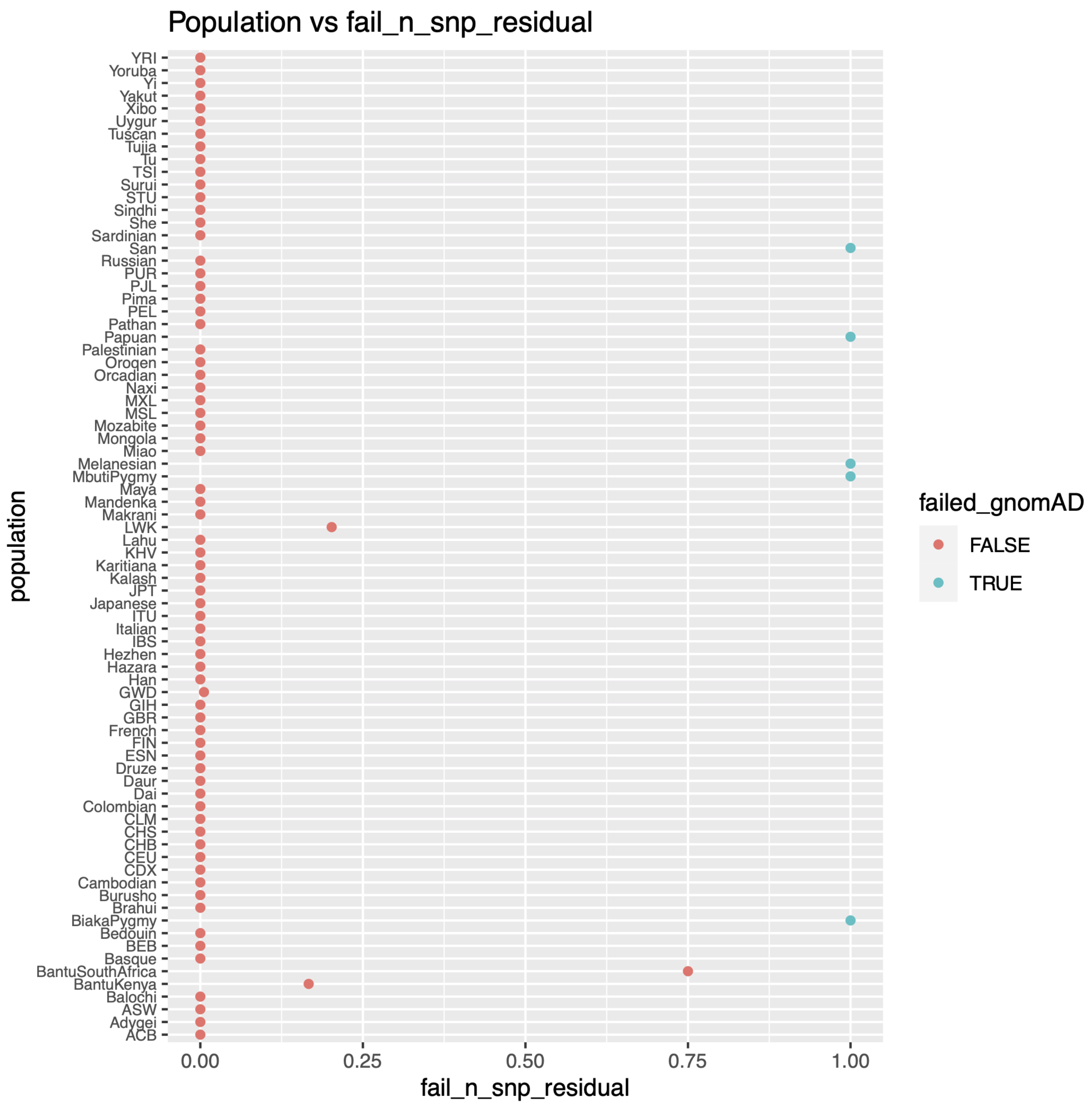

Figure S11 | Example of a filter that was included in gnomAD v3.1 but excluded from this project. The "fail_n_snp_residual" filter, which regresses out principal components from the number of SNPs in an effort to identify technical outliers, would have excluded whole continental groups and populations in this resource because these groups are distinct from the majority of individuals in gnomAD.

Analysis tutorials

To show examples of how to use the individual-level data in a cloud-computing environment, we have created a series of tutorials in iPython notebooks that make use of Hail. These tutorials show how to merge datasets, apply sample and variant QC, run ancestry analysis via PCA and visualization, generate summary statistics of

genomes by population, compute and plot population divergence statistics via F_{ST} and F_2 statistics, and intersect external datasets with this dataset and infer ancestry information using project meta-data. The organization of these notebooks is outlined in **Figure 5**.

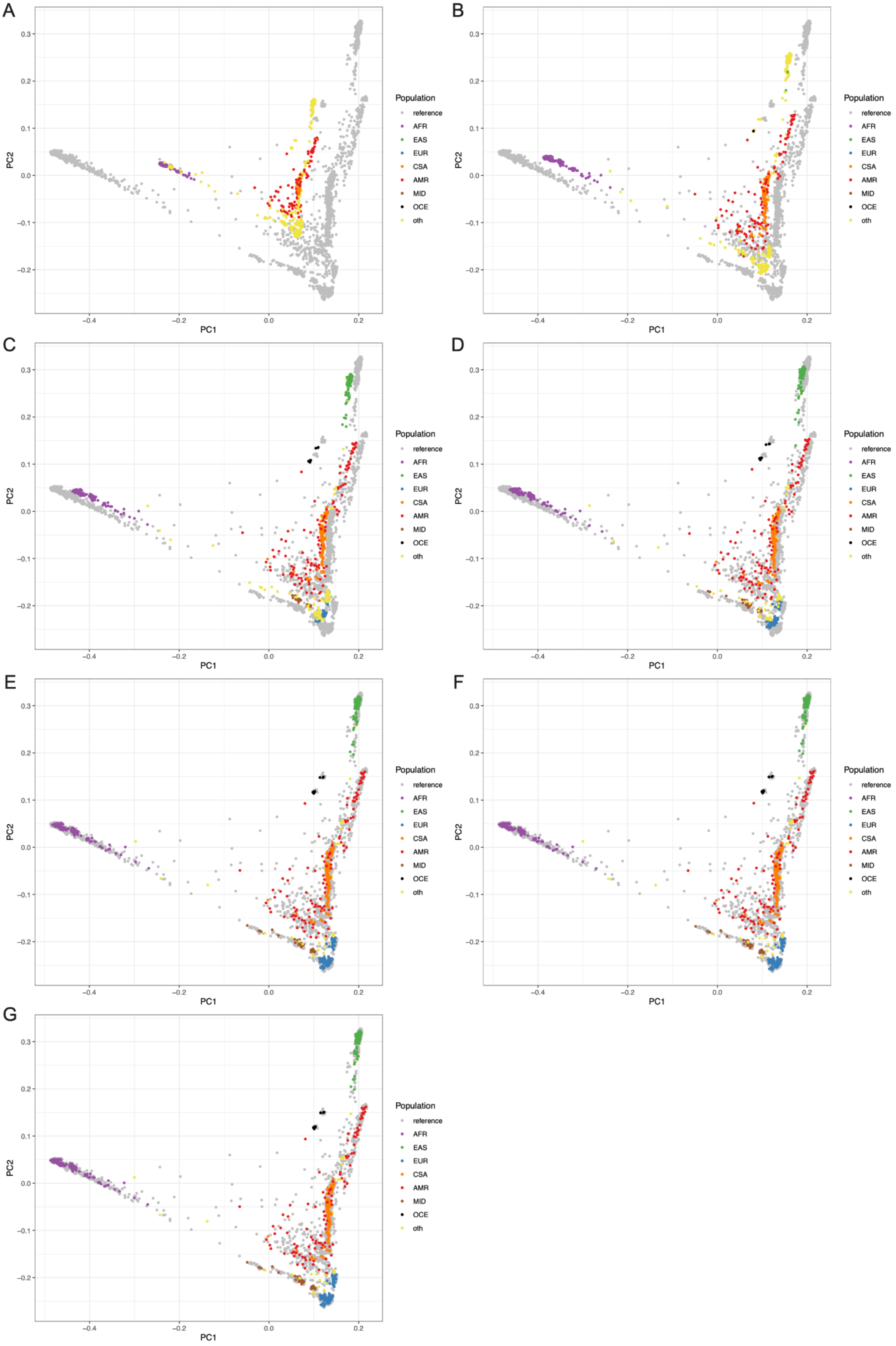
A
B
C
D
E
F
G
PC1
PC2
0.3
0.2
0.1
0.0
−0.1
−0.2
−0.4
−0.2
0.0
0.2
Population
reference
AFR
EAS
EUR
CSA
AMR
MID
OCE
oth

Figure S13 | PCA shrinkage analysis to determine acceptable levels of missingness before ancestry resolution becomes too low to accurately assign population labels. We started with a set of SNPs that were used in other PCA (e.g. **Figure 2**), which had undergone LD pruning, minor allele frequency filtering, and missingness filtering. We randomly selected 80% of samples (N=2,704) to train the random forest with corresponding meta-data labels as usual and held out 20% of samples as a test dataset (N=676). After filtering out monomorphic sites from the training dataset once samples were divided, we retained 248,634 variants which were used to train the random forest. We randomly downsampled SNPs in the test dataset to include A) 50%, B) 80%, C) 90%, D) 95%, E) 99%, F) 99.9%, and G) 100% of SNPs in the training dataset. A-G) shows the corresponding projected PCs in the test dataset, showing the extent to which shrinkage affects analyses. **Table S7** shows rates of unclassified individuals by SNP missingness in the test dataset.

Table S7 | Shrinkage analysis matches and no classification numbers by SNP missingness in the test dataset, as shown in Figure S13. There were no mismatched labels assigned.

Fraction of SNPs in test dataset out of training dataset	Match	No assignment
1	651 / 676 = 0.96	25 / 676 = 0.04
0.999	652 / 676 = 0.96	24 / 676 = 0.04
0.99	649 / 676 = 0.96	27 / 676 = 0.04
0.95	616 / 676 = 0.91	60 / 676 = 0.09
0.9	556 / 676 = 0.82	120 / 676 = 0.18
0.8	447 / 676 = 0.66	229 / 676 = 0.34
0.5	122 / 676 = 0.18	554 / 676 = 0.82

References

1. Chen, S. *et al.* A genome-wide mutational constraint map quantified from variation in 76,156 human genomes. *bioRxiv* 2022.03.20.485034 (2022) doi:10.1101/2022.03.20.485034.
2. Hail Team. *Hail*. (2021). doi:10.5281/zenodo.4504325.
3. 1000 Genomes Project Consortium *et al.* A global reference for human genetic variation. *Nature* **526**, 68–74 (2015).
4. Bergström, A. *et al.* Insights into human genetic variation and population history from 929 diverse genomes. *Science* **367**, (2020).
5. Li, H. *et al.* A synthetic-diploid benchmark for accurate variant-calling evaluation. *Nat. Methods* **15**, 595–597 (2018).
